# An induced pluripotent stem cell-based model identifies molecular targets of vincristine neurotoxicity

**DOI:** 10.1242/dmm.049471

**Published:** 2022-12-15

**Authors:** Neng-Wei Tsai, Cheng-Chen Lin, Ti-Yen Yeh, Yu-An Chiu, Hsin-Hui Chiu, Hsiang-Po Huang, Sung-Tsang Hsieh

**Affiliations:** ^1^ Department of Anatomy and Cell Biology, National Taiwan University College of Medicine, Taipei 100, Taiwan; ^2^ Department of Medical Genomics and Proteomics, National Taiwan University College of Medicine, Taipei 100, Taiwan; ^3^ Department of Pediatrics, National Taiwan University Children's Hospital, Taipei 100, Taiwan; ^4^ Department of Brain and Mind Sciences, National Taiwan University College of Medicine, Taipei 100, Taiwan; ^5^ Department of Neurology, National Taiwan University Hospital, Taipei 100, Taiwan

**Keywords:** IPSC, Sensory neuron, Nerve degeneration, Vincristine, MAP kinase, Autophagy

## Abstract

To model peripheral nerve degeneration and investigate molecular mechanisms of neurodegeneration, we established a cell system of induced pluripotent stem cell (iPSC)-derived sensory neurons exposed to vincristine, a drug that frequently causes chemotherapy-induced peripheral neuropathy. Sensory neurons differentiated from iPSCs exhibit distinct neurochemical patterns according to the immunocytochemical phenotypes, and gene expression of peripherin (PRPH, hereafter referred to as Peri) and neurofilament heavy chain (NEFH, hereafter referred to as NF). The majority of iPSC-derived sensory neurons were PRPH positive/NEFH negative, i.e. Peri(+)/NF(−) neurons, whose somata were smaller than those of Peri(+)/NF(+) neurons. On exposure to vincristine, projections from the cell body of a neuron, i.e. neurites, were degenerated quicker than somata, the lethal concentration to kill 50% (LC_50_) of neurites being below the LC_50_ for somata, consistent with the clinical pattern of length-dependent neuropathy. We then examined the molecular expression in the MAP kinase signaling pathways of, extracellular signal-regulated kinases 1/2 (MAPK1/3, hereafter referred to as ERK), p38 mitogen-activated protein kinases (MAPK11/12/13/14, hereafter referred to as p38) and c-Jun N-terminal kinases (MAPK8/9/10, hereafter referred to as JNK). Regarding these three cascades, only phosphorylation of JNK was upregulated but not that of p38 or ERK1/2. Furthermore, vincristine-treatment resulted in impaired autophagy and reduced autophagic flux. Rapamycin-treatment reversed the effect of impaired autophagy and JNK activation. These results not only established a platform to study peripheral degeneration of human neurons but also provide molecular mechanisms for neurodegeneration with the potential for therapeutic targets.

## Teaser

**Summary:** We established a platform of neurodegeneration with iPSC-derived sensory neurons and investigated the connection between neurotoxicity and signaling pathways. Our results provide the potential for therapeutic targets in vincristine-induced neurodegeneration.

## INTRODUCTION

Degeneration of sensory neurons results in peripheral neuropathy, which is common after chemotherapy; in dysmetabolic syndromes, such as diabetes; and in painful nerve degenerative diseases (Bráz and Basbaum, 2010; Freeman et al., 2020; Gewandter et al., 2019; Kobayashi and Zochodne, 2018). Although sensory neuropathy can be studied in animal models of diabetes (Frank et al., 2019; Jolivalt et al., 2016), chemotherapy (Bruna et al., 2020; Staff et al., 2020) and painless versus neuropathic pain (Currie et al., 2019; Hama and Takamatsu, 2016; Monteiro et al., 2019; Terada and Kawabata, 2015), it is crucial to conduct experiments in human systems. This is because of the differences in genetic background and molecular expressions in animals and humans (Jin et al., 2021) and, in particular, for establishing platforms to study mechanisms and to develop new therapeutic strategies (Lee-Kubli and Calcutt, 2014; Staff et al., 2020). Induced pluripotent stem cells (iPSCs) offer a new approach, fulfilling the above purposes of modeling diseases (Bianchi et al., 2018; Lee and Huang, 2017; Mertens et al., 2018) and screening drugs (Christensen et al., 2018; Fujimori et al., 2018; Stacey et al., 2018). So far, iPSCs have mainly been applied to investigate neurodegenerative diseases in the central nervous system (Lee and Huang, 2017; Stacey et al., 2018), and there have been relatively limited applications of iPSCs to study peripheral nerve degeneration (Cao et al., 2016; Little et al., 2019; Mazzara et al., 2020). When using appropriate protocols, iPSCs can be successfully converted into peripheral sensory neurons (Chambers et al., 2012), raising the opportunity to study the molecular mechanisms of neuropathy mainly influencing nociceptors.

Vincristine is a commonly used chemotherapeutic agent that targets microtubules – hence, being a main trigger of chemotherapy-induced peripheral neuropathy (Murillo and Mendes Sousa, 2018). Patients receiving vincristine also suffer from neuropathic pain, mainly attributed to injury of small-diameter sensory neurons of the nociceptive type. However, studies of vincristine on nociceptive neurons are relatively limited (Geisler, 2021; Li et al., 2020). Clinically mainly affected, are large-diameter sensory neurons, resulting, for example, in loss of proprioception (Haberberger et al., 2019), documented for nerve-conduction studies, i.e. sensory nerve physiology (McCorquodale and Smith, 2019). A crucial question is how the phenotypes of sensory neurons influence patterns of neurodegeneration, i.e. neuronopathy versus axonopathy (Jortner, 2020; McCorquodale and Smith, 2019; Valentine, 2020).

A further issue is the underlying molecular expression of neuronal injury, in particular, the MAP kinase pathway, as an important cellular response to environmental stimuli and stress (Kim and Choi, 2010). Previous studies have revealed that MAP kinases participate in neurodegenerative diseases (Ahmed et al., 2020; Obergasteiger et al., 2018). In addition, autophagy – as a mechanism for cellular homeostasis – has been implicated in neurodegenerative diseases of the brain (Corti et al., 2020; Nishijima et al., 2020; Suomi and Mcwillams, 2019). Both, the MAP kinase pathway and autophagy, are potential therapeutic targets. The above-mentioned observations raise further questions: (1) How is autophagy affected in vincristine-induced nerve degeneration and, (2) How is autophagy related to the MAP kinase signaling cascade. To address these issues, our study aimed to establish an iPSC model of vincristine-induced neurodegeneration, and investigate the molecular mechanisms and underlying signal cascades.

## RESULTS

### Nociceptive sensory neurons differentiated from human iPSCs

**Fig. 1.**
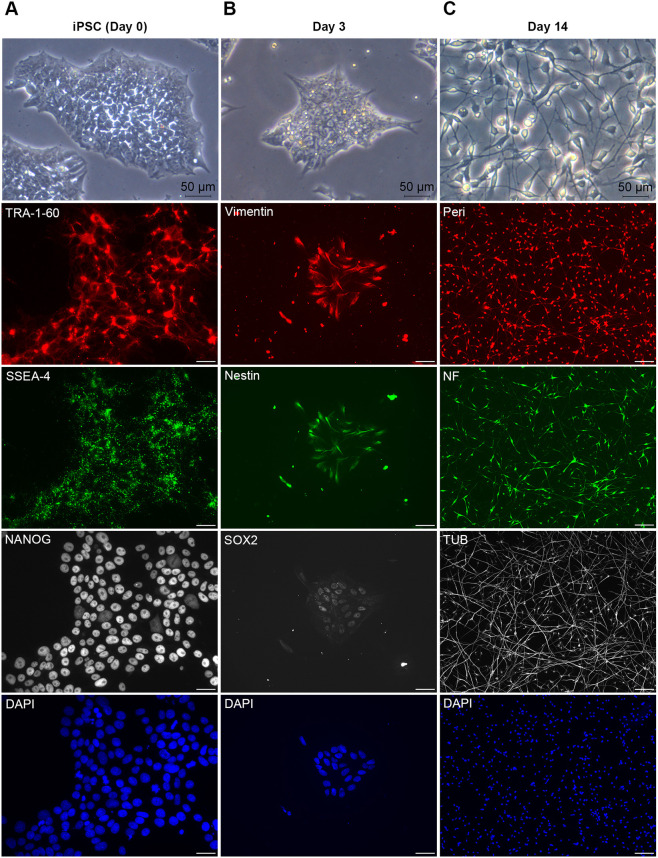
**The morphology and protein expression patterns of iPSC-derived sensory neurons during differentiation process.** Top three images: Cell morphology was observed at days 0, 3 and 14 of neuronal differentiation using bright field microscopy; scale bars: 50 µm. All other images: Differential stage markers were examined by using immunocytochemistry; scale bars: 20 µm. (A) Images of cells stained for TRA-1-60, SSEA-4 and NANOG on day 0. (B) Images of cells expressing the neural stem cell markers vimentin, nestin and SOX2 on day 3. (C) Images of differentiated neurons expressing the cytoskeletal proteins peripherin (Peri), neurofilament (NF) and βIII-tubulin (TUB) on day 14. DAPI was used to stain nuclei.

**Fig. 2.**
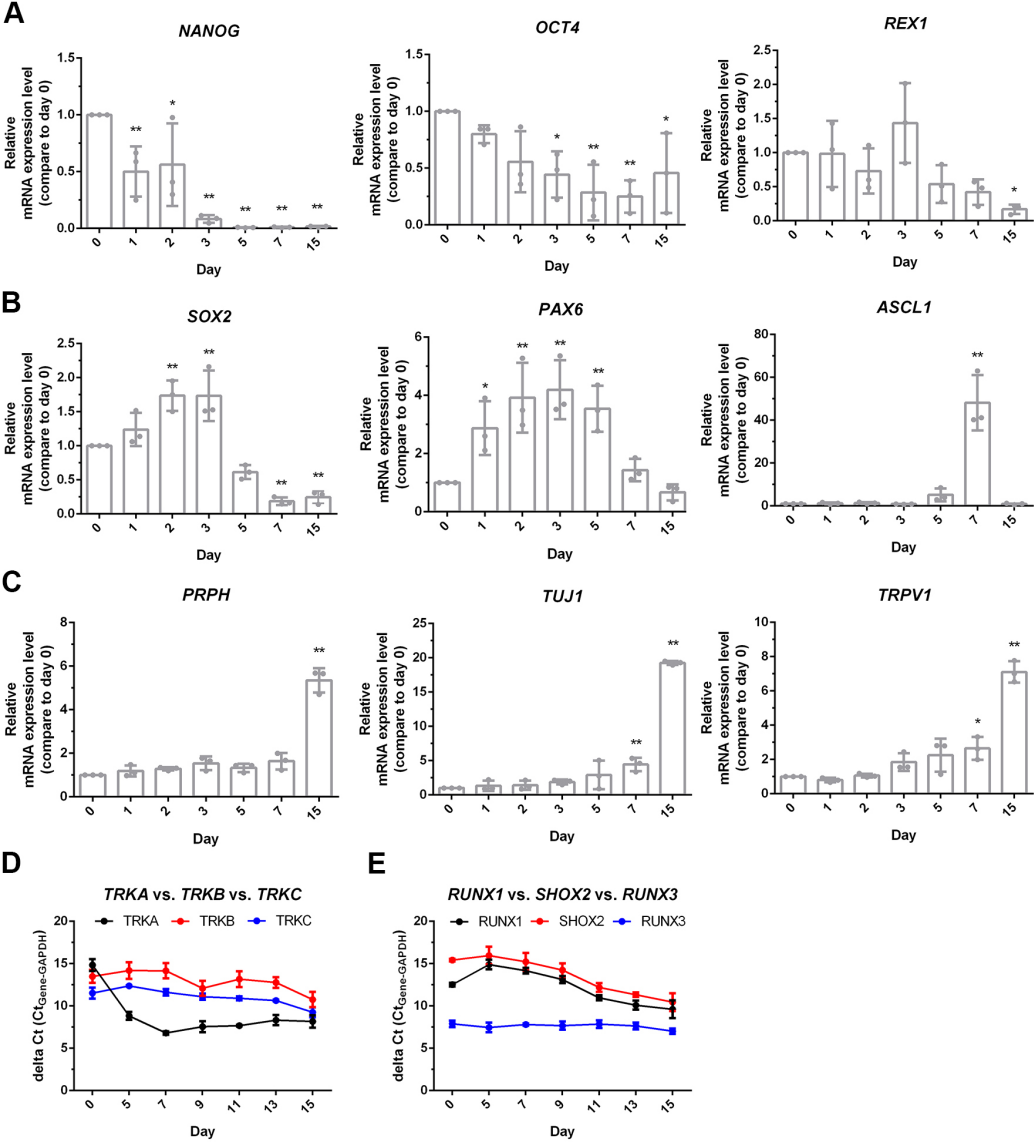
**Temporal patterns of gene expression during neuronal differentiation.** mRNA expression levels during the stages of differentiation (days 0-15) were examined using qPCR. (A) Pluripotency genes *NANOG*, *OCT4* and *REX1*. (B) Genes encoding neural stem cell marker *SOX2*, neural progenitor cell marker *PAX6* and factor of participated neuronal differentiation *ASCL1*. (C) Neuron-specific genes: *PRPH, TUJ1* and *TRPV1*. (D,E) Compared are expression levels of TRK receptors TRKA, TRKB and TRKC (D), and expression levels of transcription factors RUNX1, SHOX2 and RUNX3 (E) to distinguish between the subtype of iPSC-derived sensory neurons. mRNA expression levels are plotted as ΔCt, i.e. the difference between the Ct value of each gene in question and that of the *GAPDH* (internal control). Levels are shown relative to those on day 0. One-way ANOVA followed by Tukey's multiple comparisons test, *P<0.05, **P<0.01 compared to day 0; *n*=3.

Sensory neurons were differentiated from human iPSCs following an established protocol (see Chambers et al., 2012), using the activin receptor-like kinase (ALK) inhibitor SB431542, the selective BMP type-I receptor inhibitor LDN193189, the glycogen synthase kinase 3 (GSK3) inhibitor CHIR99021, the multi-targeted receptor tyrosine kinase (RTK) inhibitor SU5402 and the γ-secretase inhibitor DAPT that indirectly inhibits Notch. To evaluate whether the cells differentiated into sensory neurons or not, gene expression patterns and protein levels were examined using qPCR and immunocytochemistry, respectively. Protein synthesis of pluripotency markers, including homeobox protein NANOG (NANOG), podocalyxin (PODXL, hereafter referred to as TRA-1-60) and stage-specific embryonic antigen-4 (SSEA-4), was evident at the earliest stage, i.e. day 0 (Fig. 1A), confirming the pluripotency of iPSC before differentiation. At day 3 after the start of differentiation, iPSCs were induced toward the stage of neural stem cells through expression of the specific neural stem cell markers: vimentin, nestin and SOX2 (Fig. 1B). Cells expressed cytoskeletal proteins on differentiation into mature neurons: peripherin (PRPH, hereafter referred to as Peri), neurofilament heavy chain (NEFH, hereafter referred to as NF) and βIII-tubulin (TUBB3, hereafter referred to as TUB) on day 14 (Fig. 1C). At the initial stage of differentiation (day 0-2), mRNA expression levels of *NANOG*, *POU5F1* (also known as *OCT4*) and *REXO1* (also known as *REX1*) were higher than at later differentiation stages (Fig. 2A). mRNA expression of *SOX2*, *PAX6* and *ASCL1* peaked at early stages of differentiation, and decreased following the differentiation into mature neurons (day 15) (Fig. 2B). At later stages of differentiation, gene expression of *PRPH*, *TUBB3* (also known as *TUJ1*) and transient receptor potential cation channel subfamily V member 1 (*TRPV1*) was robust (Fig. 2C). These observations indicate that iPSCs were induced into sensory neurons expressing stage-specific genes and proteins.

To further assess the phenotypes of sensory neurons, we examined gene expression patterns of nociceptors, mechanoreceptors and proprioceptors, in particular, those belonging to the family of neurotrophic receptor tyrosine kinases 1, 2 and 3 (NTRK1, NTRK2 and NTRK3, hereafter referred to *TRKA*, *TRKB* and *TRKC*, respectively). Among these genes, expression of *TRKA* markedly increased (a reduced ΔCt value represents increased mRNA expression) during neuronal differentiation, whereas those of *TRKB* and *TRKC* remained relatively unchanged (Fig. 2D). We further examined the gene expression patterns of transcription factors pivotal for differentiation into nociceptive, mechanoreceptive and proprioceptive sensory neurons. Gene expression of transcription factor *RUNX1* and short stature homeobox 2 (*SHOX2*) were upregulated at the differentiation stage, whereas that of transcription factor *RUNX3* did not change substantially (Fig. 2E). These results provide evidence that human iPSCs were differentiated into sensory neurons of the nociceptive type.

### Phenotypes of iPSC-derived sensory neurons – Peri(+)/NF(+) versus Peri(+)/NF(−) neurons

To set up a platform for quantifying neurons and neurites, we used immunocytochemistry for Peri, NF and TUB. Peri and NF are neuron-specific intermediate filaments expressed in neurons of various diameters. TUB is a component of the microtubule networks. We then quantified neuron numbers, neurite lengths and neuronal soma sizes. There were two types of neuron: Peri(+)/NF(+) and Peri(+)/NF(−), making Peri suitable as a marker when staining for all neurons. In contrast to the Peri immunostaining the pattern, staining against TUB was weak at the soma but obvious at the neurite (Fig. S2). Taken together, we, therefore, chose Peri to quantify the total number of neurons and the soma size. Neurite length was best measured using immunostaining against TUB in the following experiments.

**Fig. 3.**
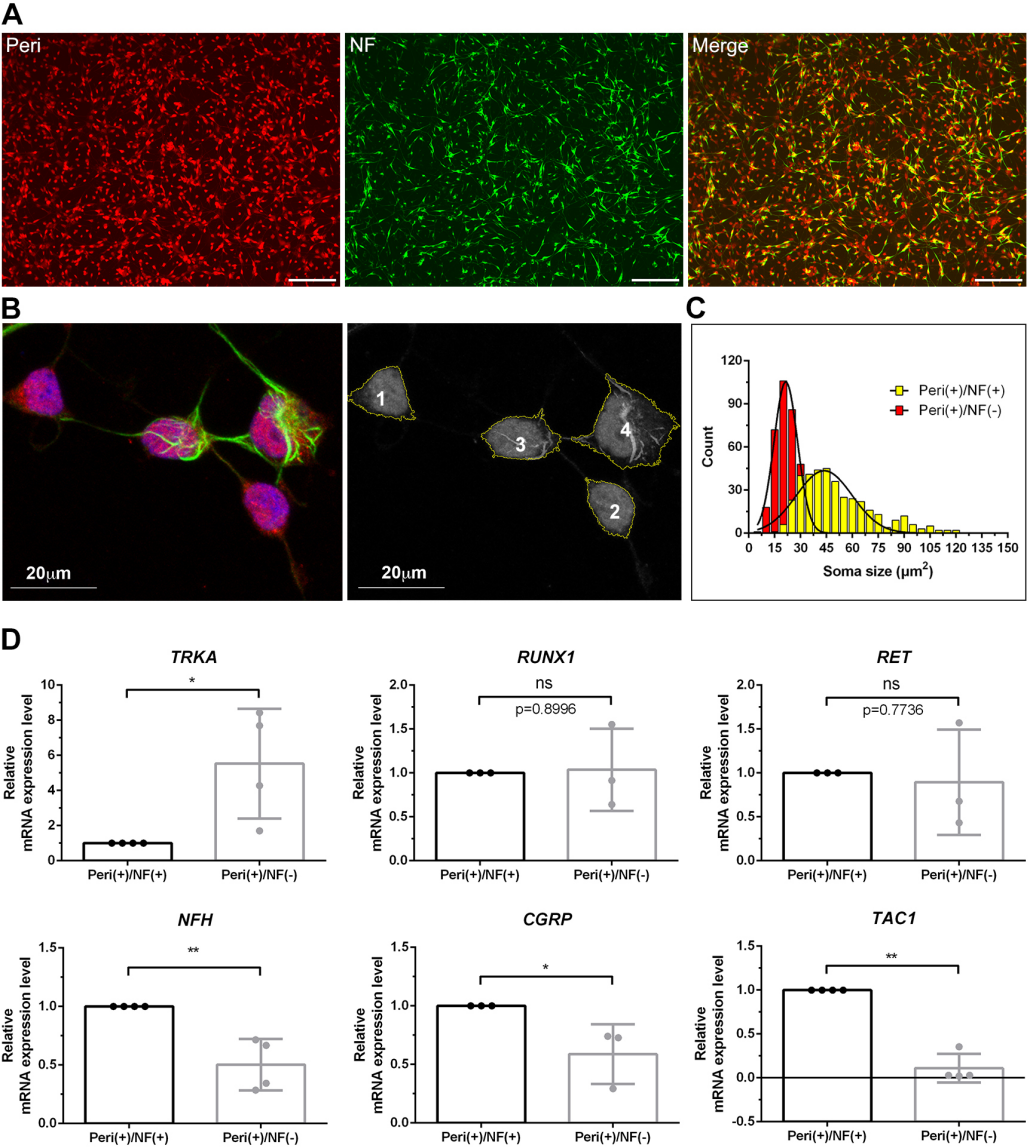
**Soma size of Peri(+)/NF(+) neurons and Peri(+)/NF(−) neurons.** iPSC-derived sensory neurons, i.e. Peri(+)/NF(+) and Peri(+)/NF(−) neurons, were stained against peripherin (Peri) and neurofilament (NF) to compare their pattern of soma size and gene expression. (A) iPSCs were differentiated into two phenotypes: Peri(+)/NF(+) (yellow) or Peri(+)/NF(−) neurons (red), scale bars: 20 µm. (B) Confocal microscopy images illustrate how neurons of both phenotype were defined. To avoid incorrectly classifying neurites as neurons, images were pre-processed by adjusting the threshold of neurite signals, i.e. neurons of both phenotypes were automatically classified by the morphometric software ImageJ, allowing measurement of soma size for each neuron. (C) Histogram showing the distribution of soma size for Peri(+)/NF(+) neurons versus Peri(+)/NF(−) neurons. Somata of Peri(+)/NF(+) neurons were larger compared with those of Peri(+)/NF(−) neurons. *n*=3. (D) iPSC-derived sensory neurons were sorted using a 4-laser FACSAriaIII cell sorter and gene expression patterns were examined by qPCR. Peri(+)/NF(−) neurons showed significantly higher expression of *TRKA* than Peri(+)/NF(+) neurons, but Peri(+)/NF(+) neurons showed significantly higher expression of *NEFH* (*NFH*) *CGRP* and *TAC1*. No significant differences were found regarding expression of *RET* and *RUNX1*. One-way ANOVA followed by Tukey's multiple comparisons test, *P<0.05 and **P<0.01 compared to Peri(+)/NF(+) neurons. *n*=3.

iPSCs were differentiated into two phenotypes according to the immunoreactivity of Peri and NF. The soma size of Peri(+)/NF(+) neurons was larger than that of Peri(+)/NF(−) neurons (Fig. 3B), i.e. the diameter of Peri(+)/NF(+) neurons was 51.2±20.86 µm (Fig. 3C, mainly ∼40-50 µm). Peri(+)/NF(−) neurons, by contrast, were smaller: 24.0±9.55 µm (mainly ∼15-30 µm, *P*<0.01), suggesting that Peri(+)/NF(−) neurons are small-diameter sensory neurons, whereas Peri(+)/NF(+) neurons are medium-sized sensory neurons.

To investigate the difference in gene expression of Peri(+)/NF(+) and Peri(+)/NF(−) neurons, cells were stained against Peri and NF, and sorted using the 4-laser FACSAriaIII cell sorter. The gene expression pattern was examined by qPCR (Fig. 3D). We found Peri(+)/NF(−) neurons to have significantly higher expression of the nociceptor gene *TRKA* than Peri(+)/NF(+) neurons but observed no difference between regarding expression of *RUNX1*. However, Peri(+)/NF(+) neurons expressed more *NEFH* (encoding neurofilament protein) and expression of the peptidergic nociceptor genes calcitonin related polypeptide alpha (CALCA, hereafter referred to as *CGRP*) and tachykinin precursor 1 (*TAC1*) were also higher. However, no significant difference in expression of the non-peptidergic nociceptive ret proto-oncogene (*RET*) was found (Fig. 3D).

### Neurotoxic effects of vincristine: neurodegeneration and phosphorylation of JNK

Clinical studies on vincristine neurotoxicity have mainly focused on large-diameter sensory neurons. The presence of neuropathic pain in patients receiving vincristine, raised the possibility of its neurotoxicity on small-diameter neurons. We, therefore, tested neurotoxicity of vincristine on iPSC-derived nociceptive neurons and calculated the lethal concentration at which 50% of them are killed (LC_50_), a measure for the degree of injury to soma and neurite.

**Fig. 4.**
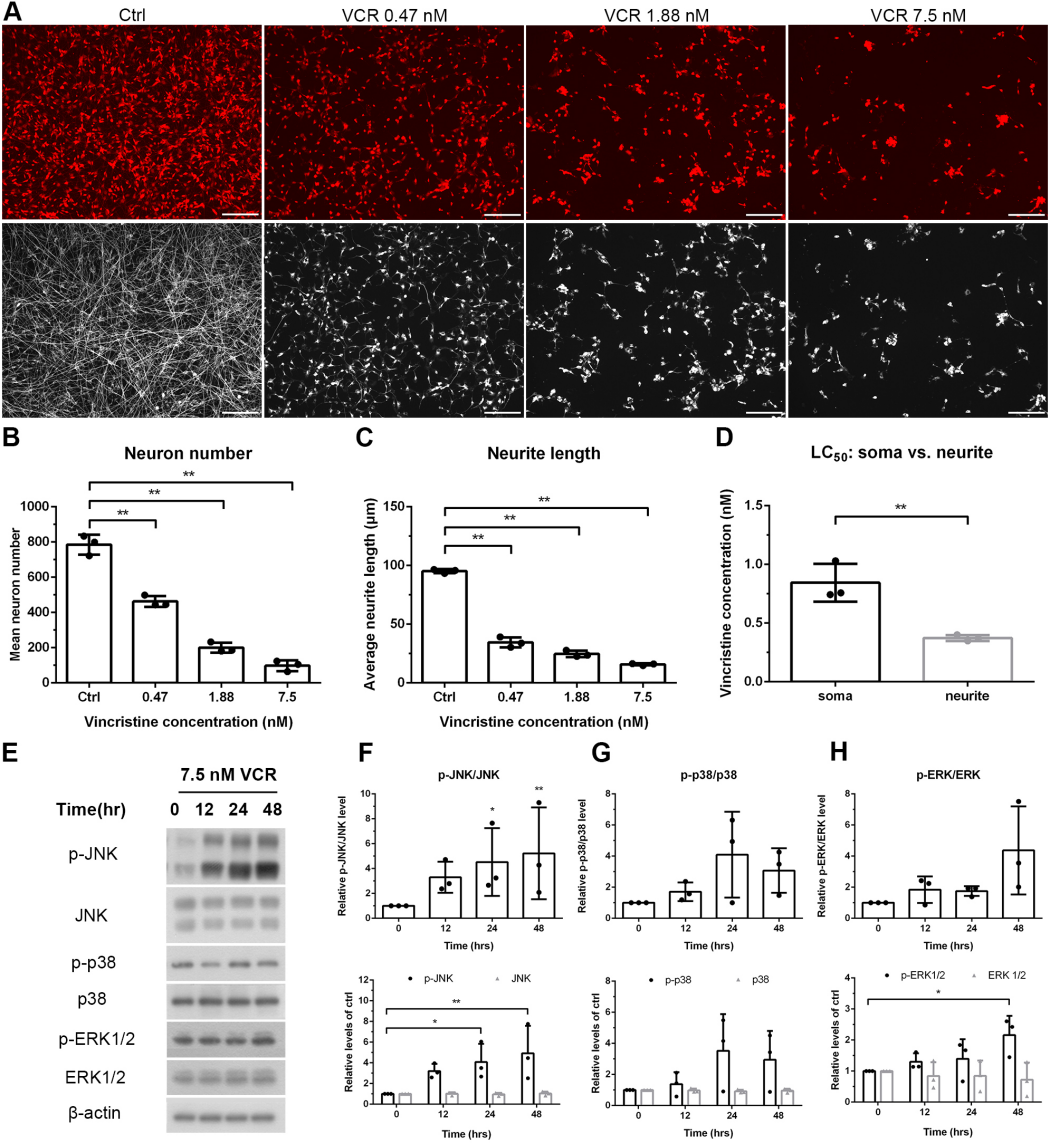
**Neurotoxic effects and activation of the MAP kinase signaling pathway (JNK, p38 and ERK) during vincristine-induced neurodegeneration.** iPSC-derived sensory neurons were treated with vincristine to investigate vincristine neurotoxicity and the effect of the drug on expression of JNK, p38 and ERK. (A) Neurons were treated for 48 h with different concentrations of vincristine (0.47, 1.88 or 7.5 nM). The neurotoxic effects of vincristine on soma versus neurite were assessed with immunostaining against peripherin (red) and βIII-tubulin (white). Scale bars: 20 µm. Images in this panel were used for quantification of neuron numbers and neurite lengths as shown in B and C, and to quantify the axon degeneration index (see Fig. S3B). The Ctrl and VCR 7.5 nM βIII-tubulin images are reused in Fig. S3A to illustrate the axon degradation analysis. (B,C) Quantitative analysis of neuron number and neurite length showed distinct pattern in soma versus neurite. (D) LC_50_ analysis, confirming that neurites are more affected by vincristine than somata. (E-H) iPSC-derived sensory neurons were treated with 7.5 nM vincristine for 0, 12, 24 and 48 h. Protein levels of JNK, p38 and ERK1/2 and their phosphorylated versions (p-JNK, p-p38, p-ERK, respectively) was assessed by western blotting; β-actin was used as an internal control. Quantification indicated a time-dependent increase after vincristine treatment only for p-JNK. One-way ANOVA followed by Tukey's multiple comparisons test, *P<0.05 and **P<0.01 compared to untreated cells, *n*=3.

Cells were treated for 48 h with vincristine at ascending concentrations (0.47 nM, 1.88 nM or 7.5 nM). There was a dose-dependent reduction of neurites, neurons and axon degeneration index (Fig. 4A-C, Fig. S3). Furthermore, reduction of neurites was more robust than loss of neuronal cell bodies. The LC_50_ of vincristine for neurites was below that for neurons (Fig. 4D, 0.37±0.025 nM vs 0.84±0.162 nM, *P*<0.01), suggesting that neurites are more vulnerable to vincristine than neurons.

We next investigated the effect of vincristine on the expression of signaling molecules. The mitogen-activated protein kinases cascade contains three main protein subfamilies, i.e. JNK, p38 and ERK. Their respective signaling pathways are activated during various physiological processes, including cell growth, cell differentiation, death, autophagy and neurodegenerative diseases (Lee and Kim, 2017; Zhou et al., 2015). Among these three pathways, only the phosphorylated form of JNK (P-JNK) achieved a time-dependent increase after treatment with vincristine (Fig. 4E-H), indicating that phosphorylation of JNK is related to vincristine neurotoxicity.

### Effect of vincristine on autophagy: autophagosome accumulation due to impaired autophagy

**Fig. 5.**
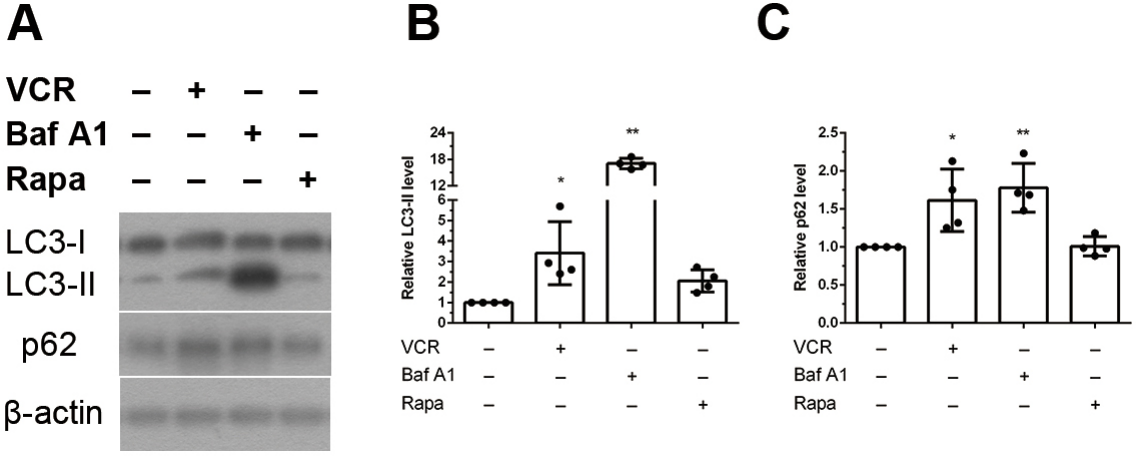
**The effect of vincristine on autophagy.** Effect of vincristine on protein levels of the autophagy-related molecules membrane-bound lipidated MAP1LC3A and SQSTM1 (LC3-II and p62, respectively) were examined with western blotting. iPSC-derived sensory neurons were treated for 24 h with 7.5 nM vincristine, the autophagy inducer rapamycin (200 nM) or the autophagy blocker bafilomycin A1 (10 nM), or were left untreated (−), and levels of cytoplasmic MAP1LC3A (LC3-I), LC3-II and p62 were compared. (A) Western blot showing that LC3-II and p62 protein levels were increased after vincristine and bafilomycin A1 treatment compared with levels in the untreated group (control group). (B,C) Quantitative analysis of the western blotting experiment. Normalization to levels of β-actin confirmed the increase of LC-II (A) and p62 (B) in vincristine- and bafilomycin A1-treated cells, indicating that autophagy was impaired after vincristine treatment. One-way ANOVA followed by Tukey's multiple comparisons test, *P<0.05 and **P<0.01 compared to untreated group (control group), *n*=4.

To understand the effects of vincristine on autophagy, we analyzed the patterns of autophagy pathway proteins. For this, we treated human iPSCs-derived sensory neurons with vincristine and analysed the levels of lipidated microtubule associated protein 1 light chain 3 alpha (MAP1LC3A, hereafter referred to as LC3-II) and sequestosome 1 (SQSTM1; hereafter referred to as p62) (Fig. 5). LC3-II and p62 protein levels following vincristine treatment were then compared to those following treatment with the autophagy inducer rapamycin and the autophagy inhibitor bafilomycin A1, a vacuolar H^+^-ATPase inhibitor that inhibits the fusion of autophagosomes with lysosomes and blocks the acidification of the lysosome, resulting in the accumulation of LC3-II (Wang et al., 2017, 2021). After 24 h of treatment with vincristine, bafilomycin A1 or rapamycin, protein levels of LC3-II and p62 were increased in the vincristine and bafilomycin A1 groups compared to those in the control group. However, there was no change in the rapamycin group (Fig. 5A-C). These results demonstrated that vincristine has an autophagy inhibitory effect on iPSCs-derived sensory neurons, similar to the effect of bafilomycin A1. Meanwhile, cleaved – i.e. activated – caspase-3, which activates downstream proteins to execute apoptosis, was not detected after vincristine treatment that indicated apoptosis did not occur in this cell model (Fig. S4).

**Fig. 6.**
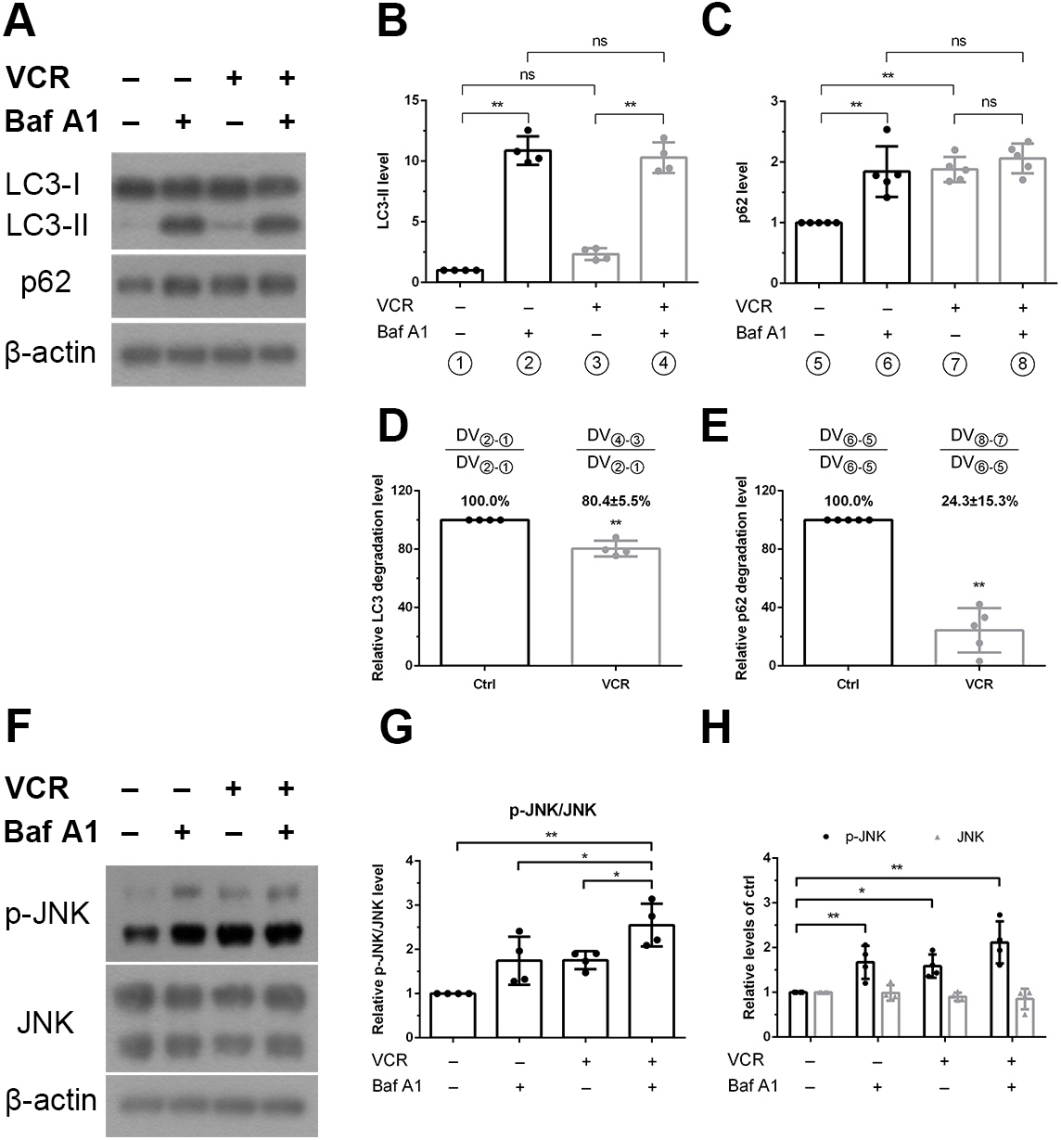
**Effects of vincristine on autophagy and the MAP kinase signaling pathway.** iPSC-derived sensory neurons were treated with vincristine to assess the relationship between autophagy and JNK protein levels. (A-C) Degradation of LC3 and p62 was determined in a turnover assay by adding bafilomycin A1 (Baf A1). Neurons were treated with 7.5 nM vincristine in the presence and absence of 10 nM bafilomycin A1 for 12 h. Following this, the differences in LC3 or p62 densitometric levels (DV) of the vincristine-treated groups – i.e. (④-③) or (⑧-⑦), respectively – were compared with that of the matching control groups – i.e. (②-①) or (⑥-⑤), respectively. (D,E) Differences in LC3-II and p62 protein levels in the presence or absence of bafilomycin A1 were normalized to protein levels in untreated neurons (control group), and are shown as relative degeneration levels. Relative degradation of p62 was markedly reduced after vincristine treatment. (F-H) Levels of phosphorylated JNK (p-JNK) were examined by western blotting; β-actin was used as an internal control. In the presence of bafilomycin A1, p-JNK levels in vincristine-treated sensory neurons were higher compared with those without bafilomycin A1. One-way ANOVA followed by Tukey's multiple comparisons test, *P<0.05 and **P<0.01 compared to untreated (control) cells, *n*=4.

Both induction and downstream blocking of autophagy can increase the number of autophagosomes. To clarify this issue, we performed turnover assays of LC3 and p62 to measure the autophagic flux, i.e. the degradation, of these proteins. Protein levels were then analysed by western blotting to examine their expression patterns in the presence and absence of 10 nM bafilomycin A1. The differences in the densitometric levels of LC3-II and p62 represented the amount of LC3-II or p62 delivered to lysosomes for degradation, i.e. the autophagic flux, which was measured by comparing the protein levels between control and treatment groups (Fig. 6A-E). The difference in the densitometric levels of the vincristine group was 80.4±5.5% [(④-③)÷(②-①), *P*<0.01; (④-③) and (②-①) denote the respective differences in densitometric levels (DVs) of LC3-II in the vincristine group or control group respectively.] of the control group (Fig. 6B,D). Furthermore, by using the same definition to monitor p62 protein levels, the degree of p62 degradation was found to be reduced in the vincristine group [(⑧-⑦)÷(⑥-⑤)=24.3±15.3%, *P*<0.01; (⑧-⑦) and (⑥-⑤) denote the respective differences in DVs of p62 in vincristine-treated and control groups.] compared to that of the control group (Fig. 6C,E). Taken together, these data indicate that vincristine blocked LC3-II and p62 degradation, thereby causing impairment of autophagy.

We then investigated the phosphorylation of JNK after treatment of iPSC-derived sensory neurons with bafilomycin A1, to assess whether the MAPK pathway has a role in autophagy. JNK phosphorylation levels were unaltered after bafilomycin A1 or vincristine treatment for 12 h. However, administration of vincristine and bafilomycin A1 together did induce phosphorylation of JNK compared with levels in the control group (Fig. 6F-H). Whereas addition of the autophagy inhibitor bafilomycin A1 to vincristine-treated cells increased levels of P-JNK, addition of the autophagy inducer rapamycin decreased them, meaning P-JNK levels were affected when autophagy was induced or inhibited. Taken together, we speculate that phosphorylation of JNK is a downstream effect of vincristine-induced autophagy. The additive effect suggests that vincristine can impair autophagy followed by JNK phosphorylation.

### Reversal of vincristine-induced effects by rapamycin: autophagy impairment and JNK phosphorylation

**Fig. 7.**
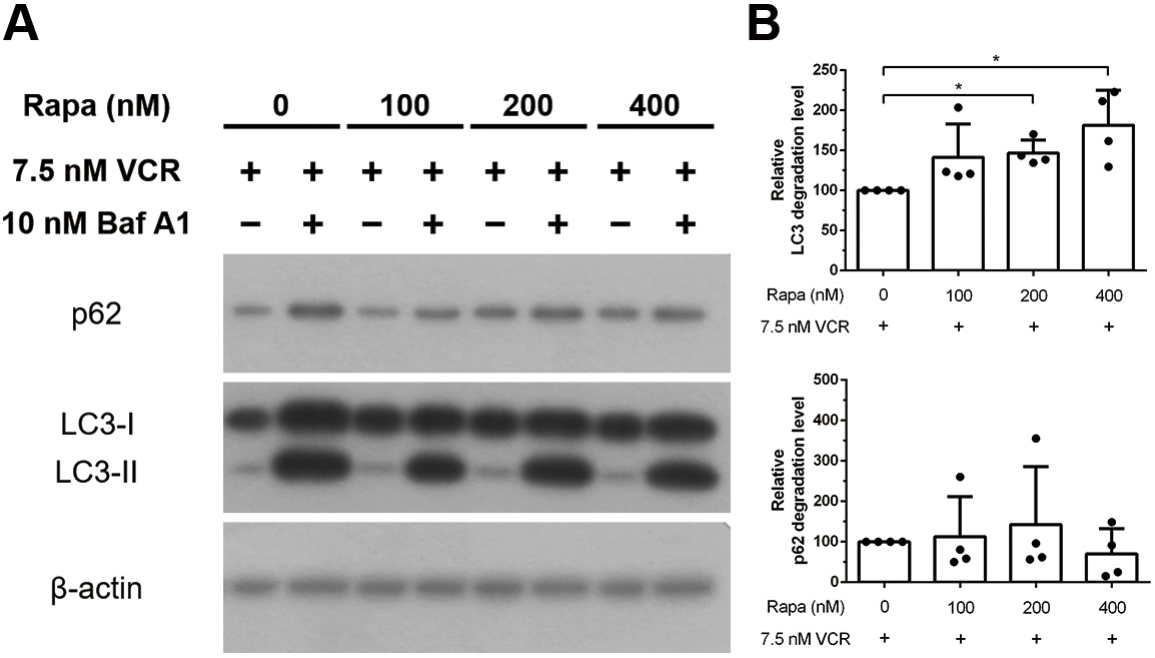
**Effect of rapamycin on vincristine-induced autophagy impairment in the presence or absence of bafilomycin A1.** iPSC-derived sensory neurons were pretreated with different concentrations (100 nM, 200 nM or 400 nM) of rapamycin for 24 h and then treated with 7.5 nM vincristine in the presence or absence of 10 nM bafilomycin A1 for 24 h. (A) Autophagy-related molecules (LC3-II and p62) were examined by western blotting with β-actin as internal control. (B) Western blot results were quantified with densitometry. Differences between neurons treated with or without bafilomycin A1 were normalized to those in the untreated rapamycin group. The results indicate that pretreatment with 200 nM or 400 nM rapamycin can reverse vincristine-induced autophagy. Paired *t*-test, *P<0.05, *n*=4.

Given that the blockade of LC3-II and the degradation of p62 resulted in autophagy impairment after treatment of iPSC-derived sensory neurons with vincristine, we tested whether the autophagy inducer rapamycin can attenuate the effect of vincristine. iPSC-derived sensory neurons were pretreated with three different concentrations of rapamycin (100 nM, 200 nM or 400 nM) for 24 h. Culture medium was washed out and replaced with 7.5 nM vincristine in new culture medium followed by addition or not of 10 nM bafilomycin for 24 h to determine autophagic flux (Fig. 7A). No significant difference between treatment groups was found regarding protein levels of p62. However, cells that had been pretreated with 200 nM or 400 nM rapamycin showed a significant increase in LC3 flux (150.7±13.36% and 198.5±5.67%, *P*<0.05, respectively) compared with cells not pre-treated with rapamycin (Fig. 7B). These results indicate that rapamycin pretreatment can alleviate the effect of vincristine-induced autophagy impairment.

**Fig. 8.**
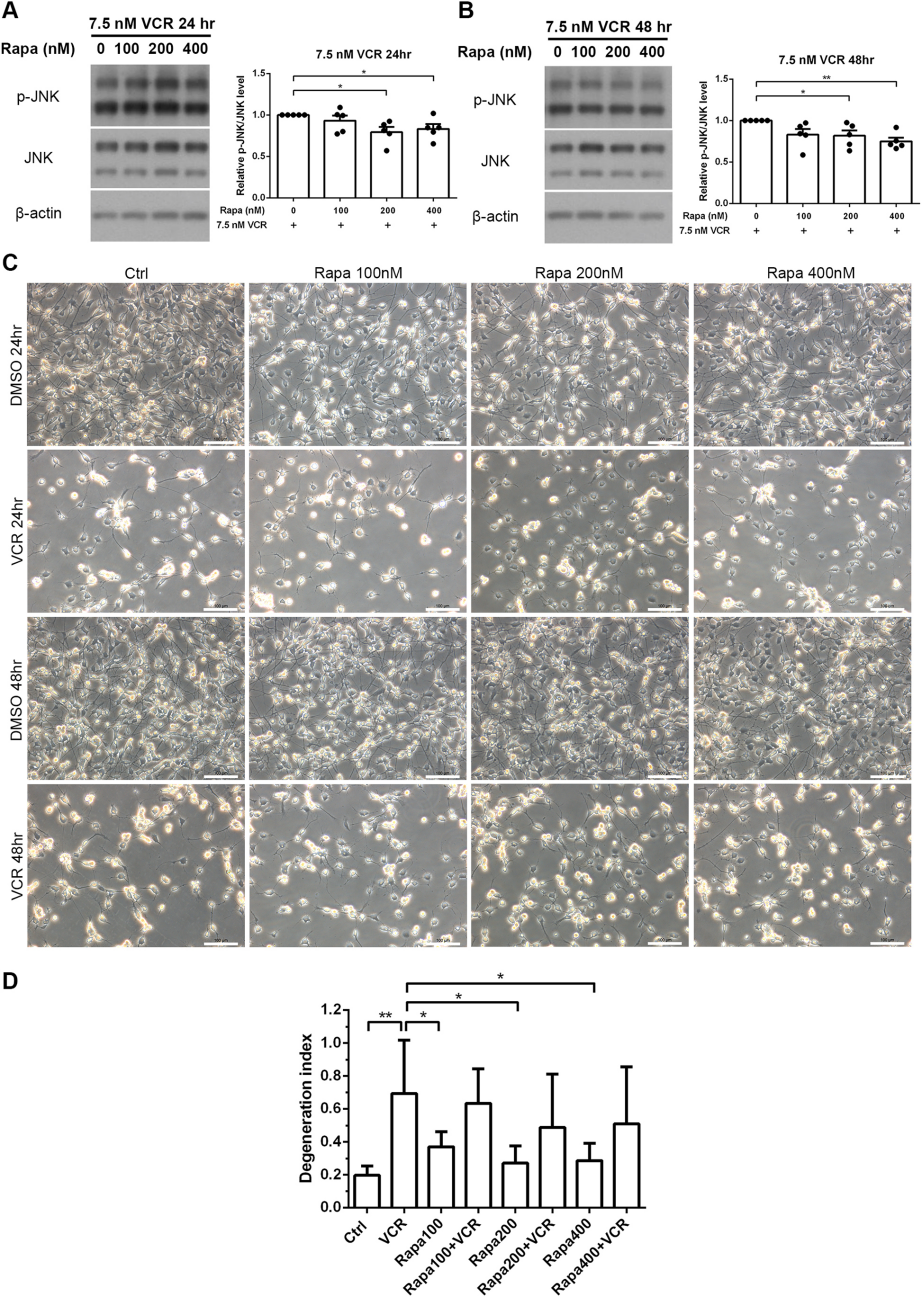
**Effects of rapamycin on vincristine-induced JNK phosphorylation.** (A,B) iPSC-derived sensory neurons were pretreated with various concentrations (100 nM, 200 nM, 400 nM) of rapamycin for 24 h, followed by treatment with 7.5 nM vincristine (VCR) for 24 h (A) and 48 h (B). Levels of phosphorylated JNK (p-JNK) were examined by western blotting; β-actin was used as an internal control, and quantified using densitometry. These results suggest that JNK phosphorylation induced by treatment with vincristine for 24 h and 48 h is significantly reduced following pretreatment with 200 nM or 400 nM rapamycin. Paired *t*-test, *P<0.05 and **P<0.01, *n*=5. (C) Bright field images, showing no difference in neuron number after pretreatment with rapamycin. Pretreatment with DMSO was used a control. (D) Degeneration index analysis, showing no difference in the neurite degeneration index after pretreatment with rapamycin at different concentrations followed by treatment with 7.5 nM vincristine for 48 h. Paired *t*-test, *P<0.05 and **P<0.01, *n*=6.

We then examined whether the effect of rapamycin extends to MAP kinases, especially to JNK. After pretreatment with different concentrations of rapamycin (0, 100, 200, 400 nM), cells were treated with 7.5 nM vincristine for 24 h or 48 h. Pretreatment with 200 nM and 400 nM rapamycin resulted in a significant reduction of phosphorylated JNK after vincristine treatment for 24 h (Fig. 8A) and 48 h (Fig. 8B). These findings suggest that rapamycin pretreatment ameliorates the effect of vincristine on phosphorylation of JNK. However, no difference was found regarding neuron number and degeneration index after treatment with rapamycin (Fig. 8C,D). We, therefore, speculate that multiple factors affect the vincristine-induced degeneration – a hypothesis that requires further investigation.

## DISCUSSION

iPSC-derived sensory neurons offer a great potential in the biomedical field. In our case, sensory neurons were differentiated from human iPSC and used as a modeling *in vitro* system to investigate CXCR4 signaling during the development of the peripheral nervous system (Terheyden-Keighley et al., 2018). iPSC-derived sensory nociceptors were induced to create a disease model of inherited erythromelalgia, providing a new strategy for pain management by regulating the voltage-gated sodium channel Na_V_1.7 (Meents et al., 2019). iPSC-derived nociceptors from patients diagnosed with small fiber neuropathy display increased excitability, which can be reversed by treatment with the FDA-approved drug lacosamide (Namer et al., 2019).

In this study, we documented (1) the profiling of iPSC, (2) different patterns of degeneration and, (3) signaling cascades that are initiated by neurodegeneration and exposure to neurotoxins. Our report successfully established a platform of peripheral sensory neurons by generating a cell model of neurodegeneration. According to the immunocytochemical pattern of Peri and NF, we differentiated iPSCs into nociceptive neurons, mainly into small- and medium-sized sensory neurons comprising both peptidergic and non-peptidergic phenotypes (Haberberger et al., 2019; Lawson et al., 2019; Nascimento et al., 2018). Differentiation was confirmed by analyzing the histograms showing frequency distributions of soma size and by using qPCR to investigate gene expression patterns. Interestingly, expression of *TRKA* increased excessively during the differentiation to sensory neurons, being 100 times higher than that of *TRKB* or *TRKC*. These findings are consistent with the functional role of TRKA in nociceptors compared to the expression of TRKB and TRKC receptors mainly for large-diameter sensory neurons of proprioceptors (Marmigère and Carroll, 2014). Thus, such a differentiation protocol provides a system to study the neurodegeneration of nociceptive neurons that contribute to the generation of neuropathic pain upon nerve injury (Hill and Bautista, 2020; Tran and Crawford, 2020; Yeh et al., 2020).

In this study, we combined (1) different neurochemical markers and (2) the calculation of LC_50_ values to decipher the susceptibility of neuronal compartments to vincristine. Given the preferential localization of soma versus neurite between Peri and TUB, we were able to compare the susceptibility of vincristine on different parts of a neuron, i.e. neurites (dentrite and axon) and soma. Intriguingly, according to the LC_50_, neurites were more vulnerable than somata to exposure of vincristine. This finding, indeed, corroborates with the clinical manifestations of vincristine neuropathy, i.e. a length-dependent distribution of symptoms, such as earlier and worse in toes compared to fingers, because axons innervating toes are longer than those innervating fingers; hence, on exposure to neurotoxins or metabolic derangements, longer axons are more vulnerable than shorter ones (Coleman and Höke, 2020; Lehmann et al., 2020). These observations raise the following topics for future elaboration: what is the molecular basis of neurite susceptibility and can it be confirmed. Additional mechanisms for vincristine-induced neurotoxicity include alteration of the bidirectional axonal transport, disruption of the transmission function of nerve impulses, damage of mitochondria, induction of neuroinflammation and inhibition of polymerization of microtubules (Fukuda et al., 2017; Starobova and Vetter, 2017; Triarico et al., 2021; Yang et al., 2021). Despite the disruption of microtubules leading to degeneration of neurites, as observed in our cell model, possible secondary effects cannot be completely excluded and require further experimentation. We demonstrated activation of the JNK signaling pathway on exposure to vincristine, and MAPK signaling pathways have a key role in neurodegenerative diseases, such as Parkinson disease, Alzheimer disease and amyotrophic lateral sclerosis (Jin and Zheng, 2019; Obergasteiger et al., 2018). They are potential therapeutic targets for neurodegenerative diseases of the central nervous system (Ahmed et al., 2020).

Phosphorylation of JNK leads to expression of pro-apoptotic molecules and, hence, cell death (Bekker et al., 2021; Uzdensky, 2019). The alterations of JNK signaling pathway associated with the neurodegeneration in *Drosophila* models of amyotrophic lateral sclerosis and Alzheimer disease (Gogia et al., 2020; Irwin et al., 2020; Yarza et al., 2015). In Alzheimer disease, JNK phosphorylation results in phosphorylation of Tau (officially known as MAPT) and impaired fast axonal transport (Morris et al., 2021). Dual leucine kinase (MAP3K12) and leucine zipper-bearing kinase (MAP3K13) both induce JNK phosphorylation, thereby leading to degeneration of cerebellar Purkinje cells through apoptotic cell death (Li et al., 2021). Furthermore, members of the Ste20 family of serine/threonine kinase activate JNK and drive neurodegeneration. Inhibition of these kinases might be a useful therapeutic target (Larhammar et al., 2017). In addition to its effect on neurodegeneration, JNK is activated in a model of chronic senescence in response to chronic administration of D-galactose, an effect that is alleviated by glycine can alleviate the JNK signaling cascade, D-galactose-induced oxidative stress neuroinflammation and cognitive impairment (Ullah et al., 2020). Only few studies exist on the roles and mechanisms of JNK signaling regarding the degeneration of peripheral nerves; for example, the orphan receptor death receptor 6 (officially known as TNFRSF21) coordinates with JNK to mediate nerve degeneration (Gamage et al., 2017). Our current study indicated that JNK phosphorylation is activated upon exposure to vincristine in iPSCs.

Autophagy plays a key role in maintaining cellular homeostasis and is tightly regulated by kinases, including JNK (King et al., 2021). Autophagy is blocked and JNK activated in amyotrophic lateral sclerosis; both pathways lead to motor neuron degeneration (Wu et al., 2019). Our study showed impaired autophagy on exposure to vincristine, with two lines of evidence: (1) increased p62 and (2) reduced autophagic flux. This is in contrast to the observation that suppression of autophagy and JNK activation can reduce injury-induced neuropathic pain (Larhammar et al., 2017). Our current study indicated that vincristine impaired autophagy and, sequentially, induced JNK phosphorylation. These results provide new insights for the development of therapies treating vincristine-induced neurodegeneration.

## MATERIALS AND METHODS

### Cell culture and differentiation

Human iPSCs were obtained from healthy donors from National Taiwan University Hospital (NTUH 201504052RINC) after informed consents were obtained (Fig. S1). This project was approved by the Ethic Committee of National Taiwan University Hospital, Taipei, Taiwan (Huang et al., 2020). Cells were maintained in Essential 8 medium supplemented with 1% penicillin-streptomycin (100×, Gibco, New York, NY), 2% Essential 8 Supplement (Gibco), Essential 8 Basal Medium (Gibco), on 3.5-cm dishes. Neuronal differentiation was started at 90-100% cell confluency following the protocol by Chambers et al. (2012). iPSCs were cultured on 3.5 cm dishes coated with Matrigel (day 0) [DMEM F-12, diluted 1:100; Corning, New York, NY). The next day (day 1), medium was replaced with knockout serum replacement (KOSR) medium with added SMAD inhibitors the activin receptor-like kinase (ALK) inhibitor SB431542 (10 µM, Selleckchem, Houston, TX) and the selective BMP type-I receptor inhibitor LDN193189 (100 nM, BioGems, Westlake Village, CA). The KOSR medium contained 15% knockout serum replacement (Gibco), 1% GlutaMAX (100×, Gibco), 1% penicillin-streptomycin (100×, Gibco), 1% non-essential amino acids (100%, Gibco), 100 µM β-mercaptoethanol (Gibco), Knockout DMEM (Gibco). Between days 3 and 10, KOSR medium was gradually changed to N2 medium [1% GlutaMAX (100×, Gibco), 1% penicillin-streptomycin (100×, Gibco), 1% N-2 supplement (100×, Gibco), 2% B-27 supplement (50×, Gibco), Neurobasal medium (Gibco)]. At the same time, the following small-molecule inhibitors were added: the glycogen synthase kinase 3 (GSK3) inhibitor CHIR99021 (3 µM, BioGems), the multi-targeted receptor tyrosine kinase (RTK) inhibitor SU5402 (10 µM, Tocris Bioscience, Minneapolis, MN) and the γ-secretase inhibitor DAPT (10 µM, Sigma, St Louis, MO), which indirectly inhibit Notch. SMAD inhibitors were removed on day 6 (CHIR99021) or day 11 (SU5402 and DAPT). Cells were passaged on day 3 and replated onto 10 mm coverslips (1.5×10^4^ cells/coverslip) coated with Matrigel by TrypLE (Gibco) on day 11 in N2 medium. On day 11, SU5402 and DAPT were depleted, and the following compounds were included in N2 medium up to day 14: CHIR99021 and human recombinant proteins: neurotrophin-3 (NT-3, 25 ng/ml, BioGems), brain-derived neurotrophic factor (BDNF, 25 ng/ml, BioGems), glial cell-derived neurotrophic factor (GDNF, 25 ng/ml, BioGems), and nerve growth factor (NGF, 25 ng/ml, Sigma). Medium was replaced with fresh one on days 13 and 15. We followed the protocol by Chambers et al. to differentiate neurons within 15 days of differentiation (Chambers et al., 2012). Characterization of the mature sensory neurons expressing markers (NF, TUB and TRPV1) was examined by qPCR during the 15-day differentiation (Chambers et al., 2012). The qPCR results revealed that these molecules were highly expressed on day 15. Differentiation protocols with similar time course were established and the cells were defined as mature sensory neurons. Furthermore, the high-efficiency protocol was gradually developed to induce iPSCs into functional sensory neurons and contributed to *in vitro* modeling and regenerative therapy (Cai et al., 2017; Terheyden-Keighley et al., 2018). Gene expression levels of *NANOG*, *OCT4*, *SOX2*, *PPRPH*, *TUJ1*, *TRPV1*, *TRKA*, *TRKB* and *TRKC* in these three clones indicated that clonal and differentiation variations did not exist in the experimental set-up used here (Fig. S1).

### Vincristine

Vincristine (1 mg, Sigma) was dissolved in 108.3377 ml DMSO as a stock solution (10 μM), and then further diluted in medium to 7.5 nM, 1.88 nM or 0.48 nM. Human iPSC-derived sensory neurons were incubated with vincristine of different concentrations with an approximately four-fold increase (0.48 nM, 1.88 nM and 7.5 nM) or solution DMSO as the control group in N2 medium on day 12.

### Immunocytochemistry

Cells were fixed with 4% paraformaldehyde for 30 min at room temperature, permeabilized with 0.5% Triton X-100 (Sigma) in PBS for 30 min, followed by incubation with primary antibodies (Table S2) in 0.5% Triton X-100 in PBS and 0.25% bovine serum albumin (BSA, Sigma) for 16 h-18 h at 4°C. After rinsing with 1× PBS three times (each for 5 min), the cells were labeled with fluorochrome-conjugated secondary antibodies diluted in 0.5% Triton X-100 in PBS and 0.25% BSA for 1 h at room temperature. Once secondary antibodies had been added, reactions were protected from light. Cells were rinsed with 1× PBS three times (5 min each), incubated with 4′,6-diamidino-2-phenylindole (DAPI, 0.5 µg/ml) in double-distilled H_2_O for 3 min at room temperature and rinsed again three times with 1× PBS (5 min each). Coverslips were mounted on gelatine-coated glass slides with glycerol and stored at −20°C.

### RNA extraction

Cells were lysed with 0.5 ml NucleoZOL (Macherey-Nagel, Düren, Germany), followed by addition of 0.2 ml RNase-free H_2_O (Sigma). Cell lysates were vortexed for 15 s, incubated at room temperature for 15 min and centrifuged at 12,000 ***g*** for 15 min at 4°C. Fractions (0.5 ml) were transferred to new 1.5 ml Eppendorf tubes, mixed gently with 0.5 ml 100% isopropanol and incubated at room temperature for 10 min to precipitate RNA. The solution was centrifuged at 12,000 ***g*** for 10 min at 4°C, supernatant removed and RNA pellets were washed with 0.5 ml 75% ethanol, followed by centrifugation at 8000 ***g*** for 3 min at 4°C. The supernatants were then discarded and the pellets dissolved with RNase-free H_2_O.

### Reverse transcriptase polymerase chain reaction (RT-PCR)

The protocol of RT-PCR followed the manufacturer instructions and our published report (Chen et al., 2019). After RNA extraction, 1 µg RNA was mixed gently with a reaction solution of 20 µl containing RNase-free H_2_O and reverse transcriptase (5× PrimeScript RT Master Mix, Takara, Kyoto, Japan). The solution was incubated at 37°C for 15 min and 85°C for 5 s to synthesize cDNA. The cDNA was stored at −20°C.

### Real-time polymerase chain reaction (qPCR)

A reaction mixture of 20 µl containing 10 µl 2× SensiFast SYBR Lo-ROX Mix (Bioline, London, UK), 0.8 µl 10 µM primer mix of forward and reverse primers, 7.2 µl RNase-free H_2_O and 2 µl cDNA (10× diluted) was incubated once at 95°C for 2 min, at 95°C for 5 s, followed by incubation at specific temperature according to the different genes for 20 s. The last two steps were repeated for 40 cycles. Gene expression was calculated using the 2^−ΔΔCt^ method described by Livak and Schmittgen (2001). The target gene expression (CT value) was normalized to GAPDH and relative expression was calculated as 2^−ΔΔCt^. TRKA/TRKB/TRKC and RUNX1/SHOX2/RUNX3 were compared using ΔCt values, i.e. the difference between the Ct value of the target gene and that of the housekeeping gene (*GAPDH*). Sequences of primers were listed in Table S3.

### Western blotting

Cells were twice washed with 1× PBS and lysed in 1× RIPA lysis buffer [1× protease inhibitor, 1× phosphatase inhibitor (PhosSTOP, Sigma)]. Samples were centrifuged at 21,913 ***g*** for 20 mins at 4°C and supernatants extracted, followed by normalization for protein (10 μg per lane) using a BCA protein assay (Tprobio, New Taipei, Taiwan). Proteins were separated using SDS-PAGE on 10–15% Tris-glycine gels and transferred to PVDF membranes (Merck, Kenilworth, NJ). After blocking non-specific binding sites of the membranes with 5% non-fat milk for 40 min at room temperature, membranes were incubated overnight at 4°C with primary antibodies (Table S2). Subsequently, membranes were washed and incubated with appropriate HRP-conjugated secondary antibodies for 1 h at room temperature. Signals were detected by using HRP-conjugated ECL (Tprobio). Quantification was performed using ImageJ.

### LC3 and p62 turnover assay

iPSC-derived sensory neurons were cultured in N2 medium supplemented with 7.5 nM vincristine and 10 nM bafilomycin A1 (Baf A1, Cayman, Ann Arbor, MI) or 7.5 nM vincristine only on day 12 – after neuronal differentiation – for 12 h. Expression of LC3-II and p62 in the presence and absence of Baf A1 was analyzed by western blotting. The difference in LC3-II and p62 protein expression after treatment with or without bafilomycin A1 represents the amount of degraded protein delivered to lysosomes. Based on the above, the difference in protein levels between control and Baf A1 treatment groups was compared to measure the autophagic flux.

### Cell sorting

On day 15 of the differentiation protocol, iPSCs were dissociated using 0.05% trypsin-EDTA (0.5%, Gibco), centrifuged at 200 ***g*** for 5 min and once resuspended in 1× PBS. Cells were fixed with 4% paraformaldehyde for 15 min at room temperature, permeabilized with 0.5% TritonX-100 (Sigma) in PBS for 30 min and incubated with primary antibodies against Peri and NF in 0.5% Triton X-100 in PBS and 0.25% BSA at 4°C overnight. After washing with 1× PBS, cells were labeled with fluorochrome-conjugated secondary antibodies Cy3 and Alexa Fluor 488 diluted in 0.5% Triton X-100 in PBS/0.25% BSA, and were then incubated for 1 h at room temperature. After labeling, cells were resuspended in 1× PBS and sorted according to expression of Peri and NF with 4-laser FACSAriaIII cell sorter (BD Biosciences).

### Imaging, quantification and statistical analysis

Images were taken with a microscope (Zeiss AxioImager A1 and Carl Zeiss LSM880) at magnification 100× (10× eyepiece and object lens) and 630× (10× eyepiece and 63× object lens) to capture five fields (one at the center and four at each quadrant). Images were then processed to 8-bit grayscale pictures and neuron number and neurite length were quantified with ImageJ (https://imagej.nih.gov/ij/index.html) or NeurphologyJ (https://hwangeric5.wixsite.com/erichwanglab/neurphologyj), a plugin to ImageJ. All statistical tests were performed using ANOVA followed by Tukey's multiple comparisons test or two-tailed paired *t*-test.

### Axon degeneration index

Images were first processed to 8-bit; then axons were captured by using Otsu threshold algorithms. The total area of axons was measured by using ‘analyze particle’ algorithms: size was set as 0–∞, circularity was set as 0–1.0. Circularity of particles >0.2 was defined as the fragmented axon (circularity set as 0.2–1.0). The degeneration index was calculated as the ratio of fragmented axon area over the total axon area (Kneynsberg et al., 2016; Sasaki et al., 2009).

## Supplementary Material

10.1242/dmm.049471_sup1 Supplementary information

## References

[DMM049471C1] Ahmed, T., Zulfiqar, A., Arguelles, S., Rasekhian, M., Nabavi, S. F., Silva, A. S. and Nabavi, S. M. (2020). Map kinase signaling as therapeutic target for neurodegeneration. *Pharmacol. Res.* 160, 105090. 10.1016/j.phrs.2020.105090 32707231

[DMM049471C2] Bekker, M., Abrahams, S., Loos, B. and Bardien, S. (2021). Can the interplay between autophagy and apoptosis be targeted as a novel therapy for Parkinson's disease? *Neurobiol. Aging* 100, 91-105. 10.1016/j.neurobiolaging.2020.12.013 33516928

[DMM049471C3] Bianchi, F., Malboubi, M., Li, Y., George, J. H., Jerusalem, A., Szele, F., Thompson, M. S. and Ye, H. (2018). Rapid and efficient differentiation of functional motor neurons from human iPSC for neural injury modelling. *Stem Cell Res* 32, 126-134. 10.1016/j.scr.2018.09.006 30278374

[DMM049471C4] Bráz, J. M. and Basbaum, A. I. (2010). Differential ATF3 expression in dorsal root ganglion neurons reveals the profile of primary afferents engaged by diverse noxious chemical stimuli. *Pain* 150, 290-301. 10.1016/j.pain.2010.05.005 20605331 PMC2922479

[DMM049471C5] Bruna, J., Alberti, P., Calls-Cobos, A., Caillaud, M., Damaj, M. I. and Navarro, X. (2020). Methods for in vivo studies in rodents of chemotherapy induced peripheral neuropathy. *Exp. Neurol.* 325, 113154. 10.1016/j.expneurol.2019.113154 31837318 PMC7105293

[DMM049471C6] Cai, S., Han, L., Ao, Q., Chan, Y. S. and Shum, D. K. (2017). Human induced pluripotent cell-derived sensory neurons for fate commitment of bone marrow-derived schwann cells: implications for remyelination therapy. *Stem Cells Transl. Med.* 6, 369-381. 10.5966/sctm.2015-0424 28191772 PMC5442799

[DMM049471C7] Cao, L., Mcdonnell, A., Nitzsche, A., Alexandrou, A., Saintot, P. P., Loucif, A. J., Brown, A. R., Young, G., Mis, M., Randall, A. et al. (2016). Pharmacological reversal of a pain phenotype in iPSC-derived sensory neurons and patients with inherited erythromelalgia. *Sci. Transl. Med.* 8, 335ra56. 10.1126/scitranslmed.aad7653 27099175

[DMM049471C8] Chambers, S. M., Qi, Y., Mica, Y., Lee, G., Zhang, X. J., Niu, L., Bilsland, J., Cao, L., Stevens, E., Whiting, P. et al. (2012). Combined small-molecule inhibition accelerates developmental timing and converts human pluripotent stem cells into nociceptors. *Nat. Biotechnol.* 30, 715-720. 10.1038/nbt.2249 22750882 PMC3516136

[DMM049471C9] Chen, C.-W., Wang, H.-L., Huang, C.-W., Huang, C.-Y., Lim, W. K., Tu, I.-C., Koorapati, A., Hsieh, S.-T., Kan, H.-W., Tzeng, S.-R. et al. (2019). Two separate functions of NME3 critical for cell survival underlie a neurodegenerative disorder. *Proc. Natl. Acad. Sci. USA* 116, 566-574. 10.1073/pnas.1818629116 30587587 PMC6329951

[DMM049471C10] Christensen, K., Roudnicky, F., Patsch, C. and Burcin, M. (2018). Requirements for using iPSC-based cell models for assay development in drug discovery. *Adv. Biochem. Eng. Biotechnol.* 163, 207-220. 10.1007/10_2017_23 29071405

[DMM049471C11] Coleman, M. P. and Höke, A. (2020). Programmed axon degeneration: from mouse to mechanism to medicine. *Nat. Rev. Neurosci.* 21, 183-196. 10.1038/s41583-020-0269-3 32152523 PMC8926152

[DMM049471C12] Corti, O., Blomgren, K., Poletti, A. and Beart, P. M. (2020). Autophagy in neurodegeneration: new insights underpinning therapy for neurological diseases. *J. Neurochem.* 154, 354-371. 10.1111/jnc.15002 32149395

[DMM049471C13] Currie, G. L., Angel-Scott, H. N., Colvin, L., Cramond, F., Hair, K., Khandoker, L., Liao, J., Macleod, M., Mccann, S. K., Morland, R. et al. (2019). Animal models of chemotherapy-induced peripheral neuropathy: a machine-assisted systematic review and meta-analysis. *PLoS Biol.* 17, e3000243. 10.1371/journal.pbio.3000243 31107871 PMC6544332

[DMM049471C14] Frank, T., Nawroth, P. and Kuner, R. (2019). Structure-function relationships in peripheral nerve contributions to diabetic peripheral neuropathy. *Pain* 160, S29-S36. 10.1097/j.pain.0000000000001530 31008847

[DMM049471C15] Freeman, R., Gewandter, J. S., Faber, C. G., Gibbons, C., Haroutounian, S., Lauria, G., Levine, T., Malik, R. A., Singleton, J. R., Smith, A. G. et al. (2020). Idiopathic distal sensory polyneuropathy: ACTTION diagnostic criteria. *Neurology* 95, 1005-1014. 10.1212/WNL.0000000000010988 33055271 PMC7734920

[DMM049471C16] Fujimori, K., Ishikawa, M., Otomo, A., Atsuta, N., Nakamura, R., Akiyama, T., Hadano, S., Aoki, M., Saya, H., Sobue, G. et al. (2018). Modeling sporadic ALS in iPSC-derived motor neurons identifies a potential therapeutic agent. *Nat. Med.* 24, 1579-1589. 10.1038/s41591-018-0140-5 30127392

[DMM049471C17] Fukuda, Y., Li, Y. and Segal, R. A. (2017). A mechanistic understanding of axon degeneration in chemotherapy-induced peripheral neuropathy. *Front. Neurosci.* 11, 481. 10.3389/fnins.2017.00481 28912674 PMC5583221

[DMM049471C18] Gamage, K. K., Cheng, I., Park, R. E., Karim, M. S., Edamura, K., Hughes, C., Spano, A. J., Erisir, A. and Deppmann, C. D. (2017). Death receptor 6 promotes wallerian degeneration in peripheral axons. *Curr. Biol.* 27, 890-896. 10.1016/j.cub.2017.01.062 28285993 PMC5360522

[DMM049471C19] Geisler, S. (2021). Vincristine- and bortezomib-induced neuropathies - from bedside to bench and back. *Exp. Neurol.* 336, 113519. 10.1016/j.expneurol.2020.113519 33129841 PMC11160556

[DMM049471C20] Gewandter, J. S., Gibbons, C. H., Campagnolo, M., Lee, J., Chaudari, J., Ward, N., Burke, L., Cavaletti, G., Herrmann, D. N., Mcarthur, J. C. et al. (2019). Clinician-rated measures for distal symmetrical axonal polyneuropathy: ACTTION systematic review. *Neurology* 93, 346-360. 10.1212/WNL.0000000000007974 31320471

[DMM049471C21] Gogia, N., Sarkar, A., Mehta, A. S., Ramesh, N., Deshpande, P., Kango-Singh, M., Pandey, U. B. and Singh, A. (2020). Inactivation of Hippo and cJun-N-terminal Kinase (JNK) signaling mitigate FUS mediated neurodegeneration in vivo. *Neurobiol. Dis.* 140, 104837. 10.1016/j.nbd.2020.104837 32199908 PMC9277911

[DMM049471C22] Haberberger, R. V., Barry, C., Dominguez, N. and Matusica, D. (2019). Human dorsal root ganglia. *Front. Cell Neurosci.* 13, 271. 10.3389/fncel.2019.00271 31293388 PMC6598622

[DMM049471C23] Hama, A. and Takamatsu, H. (2016). Chemotherapy-induced peripheral neuropathic pain and rodent models. *CNS Neurol. Disord. Drug Targets* 15, 7-19. 10.2174/1871527315666151110125325 26553161

[DMM049471C24] Hill, R. Z. and Bautista, D. M. (2020). Getting in touch with mechanical pain mechanisms. *Trends Neurosci.* 43, 311-325. 10.1016/j.tins.2020.03.004 32353335 PMC12213309

[DMM049471C25] Huang, C.-Y., Li, L.-H., Hsu, W.-T., Cheng, Y.-C., Nicholson, M. W., Liu, C.-L., Ting, C.-Y., Ko, H.-W., Syu, S.-H., Wen, C.-H. et al. (2020). Copy number variant hotspots in Han Taiwanese population induced pluripotent stem cell lines - lessons from establishing the Taiwan human disease iPSC Consortium Bank. *J. Biomed. Sci.* 27, 92. 10.1186/s12929-020-00682-7 32887585 PMC7487458

[DMM049471C26] Irwin, M., Tare, M., Singh, A., Puli, O. R., Gogia, N., Riccetti, M., Deshpande, P., Kango-Singh, M. and Singh, A. (2020). A positive feedback loop of Hippo- and c-Jun-amino-terminal kinase signaling pathways regulates amyloid-beta-mediated neurodegeneration. *Front. Cell Dev. Biol.* 8, 117. 10.3389/fcell.2020.00117 32232042 PMC7082232

[DMM049471C27] Jin, Y. and Zheng, B. (2019). Multitasking: dual leucine zipper-bearing kinases in neuronal development and stress management. *Annu. Rev. Cell Dev. Biol.* 35, 501-521. 10.1146/annurev-cellbio-100617-062644 31590586 PMC7015696

[DMM049471C28] Jin, H. Y., Moon, S. S. and Calcutt, N. A. (2021). Lost in translation? Measuring diabetic neuropathy in humans and animals. *Diabetes Metab. J.* 45, 27-42. 10.4093/dmj.2020.0216 33307618 PMC7850880

[DMM049471C29] Jolivalt, C. G., Frizzi, K. E., Guernsey, L., Marquez, A., Ochoa, J., Rodriguez, M. and Calcutt, N. A. (2016). Peripheral neuropathy in mouse models of diabetes. *Curr. Protoc. Mouse Biol.* 6, 223-255. 10.1002/cpmo.11 27584552 PMC5023323

[DMM049471C30] Jortner, B. S. (2020). Common structural lesions of the peripheral nervous system. *Toxicol. Pathol.* 48, 96-104. 10.1177/0192623319826068 30722748

[DMM049471C31] Kim, E. K. and Choi, E. J. (2010). Pathological roles of MAPK signaling pathways in human diseases. *Biochim. Biophys. Acta* 1802, 396-405. 10.1016/j.bbadis.2009.12.009 20079433

[DMM049471C32] King, K. E., Losier, T. T. and Russell, R. C. (2021). Regulation of autophagy enzymes by nutrient signaling. *Trends Biochem. Sci.* 1206, 67-83. 10.1007/978-981-15-0602-4_3 33593593

[DMM049471C33] Kneynsberg, A., Collier, T. J., Manfredsson, F. P. and Kanaan, N. M. (2016). Quantitative and semi-quantitative measurements of axonal degeneration in tissue and primary neuron cultures. *J. Neurosci. Methods* 266, 32-41. 10.1016/j.jneumeth.2016.03.004 27031947 PMC4874894

[DMM049471C34] Kobayashi, M. and Zochodne, D. W. (2018). Diabetic neuropathy and the sensory neuron: new aspects of pathogenesis and their treatment implications. *J. Diabetes Investig.* 9, 1239-1254. 10.1111/jdi.12833 PMC6215951 29533535

[DMM049471C35] Larhammar, M., Huntwork-Rodriguez, S., Rudhard, Y., Sengupta-Ghosh, A. and Lewcock, J. W. (2017). The Ste20 family kinases MAP4K4, MINK1, and TNIK converge to regulate stress-induced JNK signaling in neurons. *J. Neurosci.* 37, 11074-11084. 10.1523/JNEUROSCI.0905-17.2017 28993483 PMC6596808

[DMM049471C36] Lee, S. and Huang, E. J. (2017). Modeling ALS and FTD with iPSC-derived neurons. *Brain Res.* 1656, 88-97. 10.1016/j.brainres.2015.10.003 26462653 PMC4833714

[DMM049471C37] Lee, J. K. and Kim, N. J. (2017). Recent advances in the inhibition of p38 MAPK as a potential strategy for the treatment of Alzheimer's disease. *Molecules* 22, 1287. 10.3390/molecules22081287 28767069 PMC6152076

[DMM049471C38] Lee-Kubli, C. A. and Calcutt, N. A. (2014). Painful neuropathy: mechanisms. *Handb. Clin. Neurol.* 126, 533-557. 10.1016/B978-0-444-53480-4.00034-5 25410243

[DMM049471C39] Lehmann, H. C., Staff, N. P. and Hoke, A. (2020). Modeling chemotherapy induced peripheral neuropathy (CIPN) in vitro: prospects and limitations. *Exp. Neurol.* 326, 113140. 10.1016/j.expneurol.2019.113140 31812556 PMC8926153

[DMM049471C40] Li, G.-Z., Hu, Y.-H., Li, D.-Y., Zhang, Y., Guo, H.-L., Li, Y.-M., Chen, F. and Xu, J. (2020). Vincristine-induced peripheral neuropathy: a mini-review. *Neurotoxicology* 81, 161-171. 10.1016/j.neuro.2020.10.004 33053366

[DMM049471C41] Li, Y., Ritchie, E. M., Steinke, C. L., Qi, C., Chen, L., Zheng, B. and Jin, Y. (2021). Activation of MAP3K DLK and LZK in Purkinje cells causes rapid and slow degeneration depending on signaling strength. *Elife* 10, e63509. 10.7554/eLife.63509 33475086 PMC7870138

[DMM049471C42] Little, D., Ketteler, R., Gissen, P. and Devine, M. J. (2019). Using stem cell-derived neurons in drug screening for neurological diseases. *Neurobiol. Aging* 78, 130-141. 10.1006/meth.2001.1262 30925301

[DMM049471C43] Livak, K. J., and Schmittgen, T. D. (2001). Analysis of relative gene expression data using real-time quantitative PCR and the 2^−ΔΔCT^ method. *Methods* 25, 402-408. 10.1016/j.neurobiolaging.2019.02.008 11846609

[DMM049471C75] Marmigère, F. and Carroll, P. (2014). Neurotrophin signalling and transcription programmes interactions in the development of somatosensory neurons. *Handb. Exp. Pharmacol.* 220, 329-353. 10.1007/978-3-642-45106-5_13 24668479

[DMM049471C44] Mazzara, P. G., Muggeo, S., Luoni, M., Massimino, L., Zaghi, M., Valverde, P. T., Brusco, S., Marzi, M. J., Palma, C., Colasante, G. et al. (2020). Frataxin gene editing rescues Friedreich's ataxia pathology in dorsal root ganglia organoid-derived sensory neurons. *Nat. Commun.* 11, 4178. 10.1038/s41467-020-17954-3 32826895 PMC7442818

[DMM049471C45] Mccorquodale, D. and Smith, A. G. (2019). Clinical electrophysiology of axonal polyneuropathies. *Handb. Clin. Neurol.* 161, 217-240. 10.1016/B978-0-444-64142-7.00051-5 31307603

[DMM049471C46] Meents, J. E., Bressan, E., Sontag, S., Foerster, A., Hautvast, P., Rösseler, C., Hampl, M., Schüler, H., Goetzke, R., Le, T. K. C. et al. (2019). The role of Nav1.7 in human nociceptors: insights from human induced pluripotent stem cell-derived sensory neurons of erythromelalgia patients. *Pain* 160, 1327-1341. 10.1097/j.pain.0000000000001511 30720580 PMC6554007

[DMM049471C47] Mertens, J., Reid, D., Lau, S., Kim, Y. and Gage, F. H. (2018). Aging in a dish: iPSC-derived and directly induced neurons for studying brain aging and age-related neurodegenerative diseases. *Annu. Rev. Genet.* 52, 271-293. 10.1146/annurev-genet-120417-031534 30208291 PMC6415910

[DMM049471C48] Monteiro, C., Cardoso-Cruz, H. and Galhardo, V. (2019). Animal models of congenital hypoalgesia: Untapped potential for assessing pain-related plasticity. *Neurosci. Lett.* 702, 51-60. 10.1016/j.neulet.2018.11.045 30503913

[DMM049471C49] Morris, S. L., Tsai, M.-Y., Aloe, S., Bechberger, K., König, S., Morfini, G. and Brady, S. T. (2021). Defined Tau phosphospecies differentially inhibit fast axonal transport through activation of two independent signaling pathways. *Front. Mol. Neurosci.* 13, 610037. 10.3389/fnmol.2020.610037 33568975 PMC7868336

[DMM049471C50] Murillo, B. and Mendes Sousa, M. (2018). Neuronal intrinsic regenerative capacity: the impact of microtubule organization and axonal transport. *Dev. Neurobiol.* 78, 952-959. 10.1002/dneu.22602 29738096

[DMM049471C51] Namer, B., Schmidt, D., Eberhardt, E., Maroni, M., Dorfmeister, E., Kleggetveit, I. P., Kaluza, L., Meents, J., Gerlach, A., Lin, Z. et al. (2019). Pain relief in a neuropathy patient by lacosamide: proof of principle of clinical translation from patient-specific iPS cell-derived nociceptors. *EBioMedicine* 39, 401-408. 10.1016/j.ebiom.2018.11.042 30503201 PMC6354557

[DMM049471C52] Nishijima, E., Namekata, K., Kimura, A., Guo, X., Harada, C., Noro, T., Nakano, T. and Harada, T. (2020). Topical ripasudil stimulates neuroprotection and axon regeneration in adult mice following optic nerve injury. *Sci. Rep.* 10, 15709. 10.1038/s41598-020-72748-3 32973242 PMC7515881

[DMM049471C53] Obergasteiger, J., Frapporti, G., Pramstaller, P. P., Hicks, A. A. and Volta, M. (2018). A new hypothesis for Parkinson's disease pathogenesis: GTPase-p38 MAPK signaling and autophagy as convergence points of etiology and genomics. *Mol. Neurodegener.* 13, 40. 10.1186/s13024-018-0273-5 30071902 PMC6090926

[DMM049471C54] Sasaki, Y., Vohra, B. P., Lund, F. E. and Milbrandt, J. (2009). Nicotinamide mononucleotide adenylyl transferase-mediated axonal protection requires enzymatic activity but not increased levels of neuronal nicotinamide adenine dinucleotide. *J. Neurosci.* 29, 5525-5535. 10.1523/JNEUROSCI.5469-08.2009 19403820 PMC3162248

[DMM049471C55] Stacey, P., Wassermann, A. M., Kammonen, L., Impey, E., Wilbrey, A. and Cawkill, D. (2018). Plate-based phenotypic screening for pain using human iPSC-derived sensory neurons. *SLAS Discov.* 23, 585-596. 10.1177/2472555218764678 29547351

[DMM049471C56] Staff, N. P., Fehrenbacher, J. C., Caillaud, M., Damaj, M. I., Segal, R. A. and Rieger, S. (2020). Pathogenesis of paclitaxel-induced peripheral neuropathy: a current review of in vitro and in vivo findings using rodent and human model systems. *Exp. Neurol.* 324, 113121. 10.1016/j.expneurol.2019.113121 31758983 PMC6993945

[DMM049471C57] Starobova, H. and Vetter, I. (2017). Pathophysiology of chemotherapy-induced peripheral neuropathy. *Front. Mol. Neurosci.* 10, 174. 10.3389/fnmol.2017.00174 28620280 PMC5450696

[DMM049471C58] Suomi, F. and Mcwilliams, T. G. (2019). Autophagy in the mammalian nervous system: a primer for neuroscientists. *Neuronal. Signal* 3, Ns20180134. 10.1042/NS20180134 32269837 PMC7104325

[DMM049471C59] Terada, Y. and Kawabata, A. (2015). H2S and pain: a novel aspect for processing of somatic, visceral and neuropathic pain signals. *Handb. Exp. Pharmacol.* 230, 217-230. 10.1007/978-3-319-18144-8_11 26162837

[DMM049471C60] Terheyden-Keighley, D., Zhang, X., Brand-Saberi, B. and Theiss, C. (2018). CXCR4/SDF1 signalling promotes sensory neuron clustering in vitro. *Biol. Open* 7, bio035568. 10.1242/bio.035568 30135081 PMC6176946

[DMM049471C61] Tran, E. L. and Crawford, L. K. (2020). Revisiting PNS plasticity: how uninjured sensory afferents promote neuropathic pain. *Front. Cell Neurosci.* 14, 612982. 10.3389/fncel.2020.612982 33362476 PMC7759741

[DMM049471C62] Triarico, S., Romano, A., Attinà, G., Capozza, M. A., Maurizi, P., Mastrangelo, S. and Ruggiero, A. (2021). Vincristine-induced peripheral neuropathy (VIPN) in pediatric tumors: mechanisms, risk factors, strategies of prevention and treatment. *Int. J. Mol. Sci.* 22, 4112. 10.3390/ijms22084112 33923421 PMC8073828

[DMM049471C63] Ullah, R., Jo, M. H., Riaz, M., Alam, S. I., Saeed, K., Ali, W., Rehman, I. U., Ikram, M. and Kim, M. O. (2020). Glycine, the smallest amino acid, confers neuroprotection against D-galactose-induced neurodegeneration and memory impairment by regulating c-Jun N-terminal kinase in the mouse brain. *J. Neuroinflammation* 17, 303. 10.1186/s12974-020-01989-w 33059700 PMC7566050

[DMM049471C64] Uzdensky, A. B. (2019). Apoptosis regulation in the penumbra after ischemic stroke: expression of pro- and antiapoptotic proteins. *Apoptosis* 24, 687-702. 10.1007/s10495-019-01556-6 31256300

[DMM049471C65] Valentine, W. M. (2020). Toxic peripheral neuropathies: agents and mechanisms. *Toxicol. Pathol.* 48, 152-173. 10.1177/0192623319854326 31181992 PMC6901819

[DMM049471C66] Wang, Q., You, T., Fan, H., Wang, Y., Chu, T., Poncz, M. and Zhu, L. (2017). Rapamycin and bafilomycin A1 alter autophagy and megakaryopoiesis. *Platelets* 28, 82-89. 10.1080/09537104.2016.1204436 27534900

[DMM049471C67] Wang, R., Wang, J., Hassan, A., Lee, C. H., Xie, X. S. and Li, X. (2021). Molecular basis of V-ATPase inhibition by bafilomycin A1. *Nat. Commun.* 12, 1782. 10.1038/s41467-021-22111-5 33741963 PMC7979754

[DMM049471C68] Wu, C., Watts, M. E. and Rubin, L. L. (2019). MAP4K4 activation mediates motor neuron degeneration in amyotrophic lateral sclerosis. *Cell Rep.* 26, 1143-1156.e5. 10.1016/j.celrep.2019.01.019 30699345

[DMM049471C69] Yang, Q.-Y., Hu, Y.-H., Guo, H.-L., Xia, Y., Zhang, Y., Fang, W. R., Li, Y.-M., Xu, J., Chen, F., Wang, Y.-R. et al. (2021). Vincristine-induced peripheral neuropathy in childhood acute lymphoblastic leukemia: genetic variation as a potential risk factor. *Front. Pharmacol.* 12, 771487. 10.3389/fphar.2021.771487 34955843 PMC8696478

[DMM049471C70] Yarza, R., Vela, S., Solas, M. and Ramirez, M. J. (2015). c-Jun N-terminal kinase (JNK) signaling as a therapeutic target for Alzheimer's disease. *Front. Pharmacol.* 6, 321. 10.3389/fphar.2015.00321 26793112 PMC4709475

[DMM049471C71] Yeh, T.-Y., Luo, I.-W., Hsieh, Y.-L., Tseng, T.-J., Chiang, H. and Hsieh, S.-T. (2020). Peripheral neuropathic pain: from experimental models to potential therapeutic targets in dorsal root ganglion neurons. *Cells* 9, 2725. 10.3390/cells9122725 33371371 PMC7767346

[DMM049471C72] Zhou, Y. Y., Li, Y., Jiang, W. Q. and Zhou, L. F. (2015). MAPK/JNK signalling: a potential autophagy regulation pathway. *Biosci. Rep.* 35, e00199. 10.1042/BSR20140141 26182361 PMC4613668

