# Supplementary material for: An induced pluripotent stem cell-based model identifies molecular targets of vincristine neurotoxicity
Source: Dis Model Mech. 2022 Dec 15;15(12):dmm049471. doi: 10.1242/dmm.049471 (PMC10655812; doi:10.1242/dmm.049471)
Supplement: Supplementary information [file dmm-15-049471-s1.pdf]

A

Name	Type	Species	Tissue	Strain Ethnicity	Gender	Age
NTUH-iPSC-02-02	Sendai virus reprogrammed iPSC	Human	PBMC	Taiwanese	Male	52
IBMS-iPSC-02-07	Sendai virus reprogrammed iPSC	Human	PBMC	Taiwanese	Male	22
TVGH-iPSC-02-07	Sendai virus reprogrammed iPSC	Human	PBMC	Taiwanese	Male	26

B

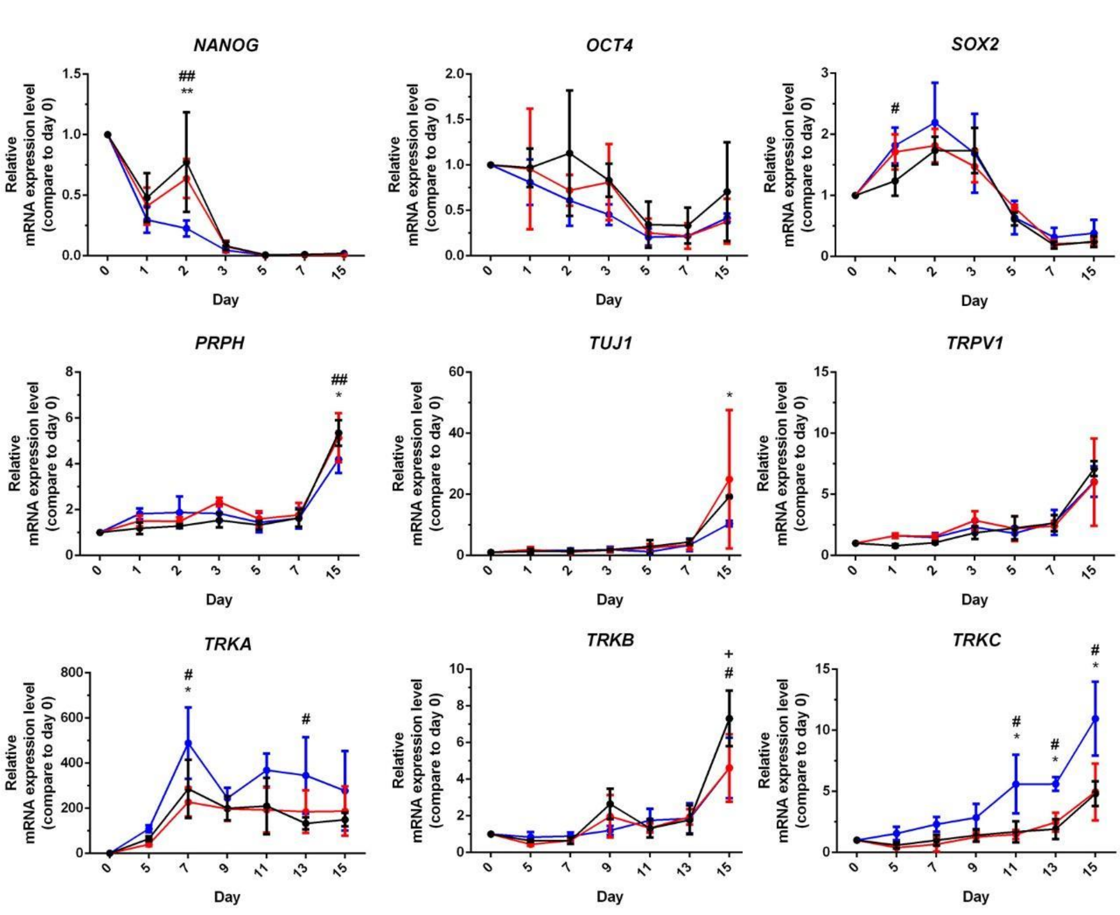

Fig. S1. Origin and gene expression pattern during differentiation of human induced pluripotent stem cells

There are three iPSC cell lines: NTUH, IBMS, and TVGH. They were differentiated into sensory neurons and then examined gene expressions by qPCR. (A) The information and source of iPSCs. PBMC: peripheral blood mononuclear cell. (B) The time course of gene expression during the stage of differentiation was examined with qPCR. All mRNA expression was normalized to GAPDH and shown as relative levels to that of day 0. One way ANOVA followed by Tukey's multiple comparisons test, # $p < 0.05$, # #p <0.01 between NTUH and IBMS; * $p < 0.05$, ** $p < 0.01$ between NTUH and TVGH; + $p < 0.05$, ++ $p < 0.01$ between IBMS and TVGH. n=3.

Disease Models & Mechanisms • Supplementary information

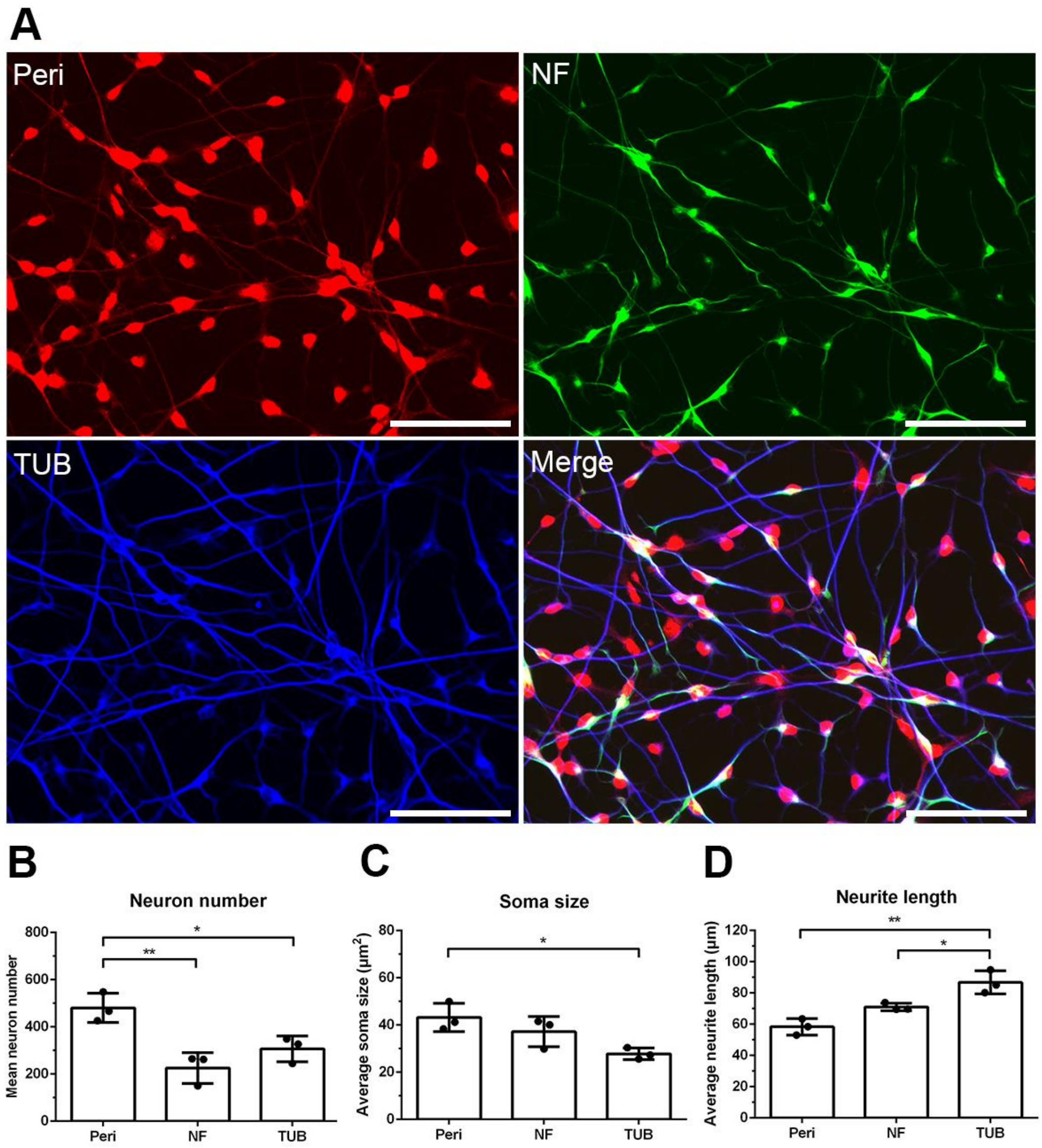

Fig. S2. Relationship of different neuronal markers with their morphometry. Differentiated neurons were immunocytochemistry stained with peripherin (Peri), neurofilament heavy chain (NF), and βIII-tubulin (TUB), then quantified with morphometry analysis. (A) There were distinct expression patterns of peripherin (Peri), neurofilament heavy chain (NF), and βIII-tubulin (TUB). Scale bar = 20 µm. (B-D) The quantitative analysis of neuron number, soma size, and neurite length by staining with peripherin, neurofilament, and βIII-tubulin, indicated that neurons have different neurochemical phenotypes with compound morphometry. One way ANOVA followed by Tukey's multiple comparisons test, * $p < 0.05$, ** $p < 0.01$, n=3.

Disease Models & Mechanisms • Supplementary information

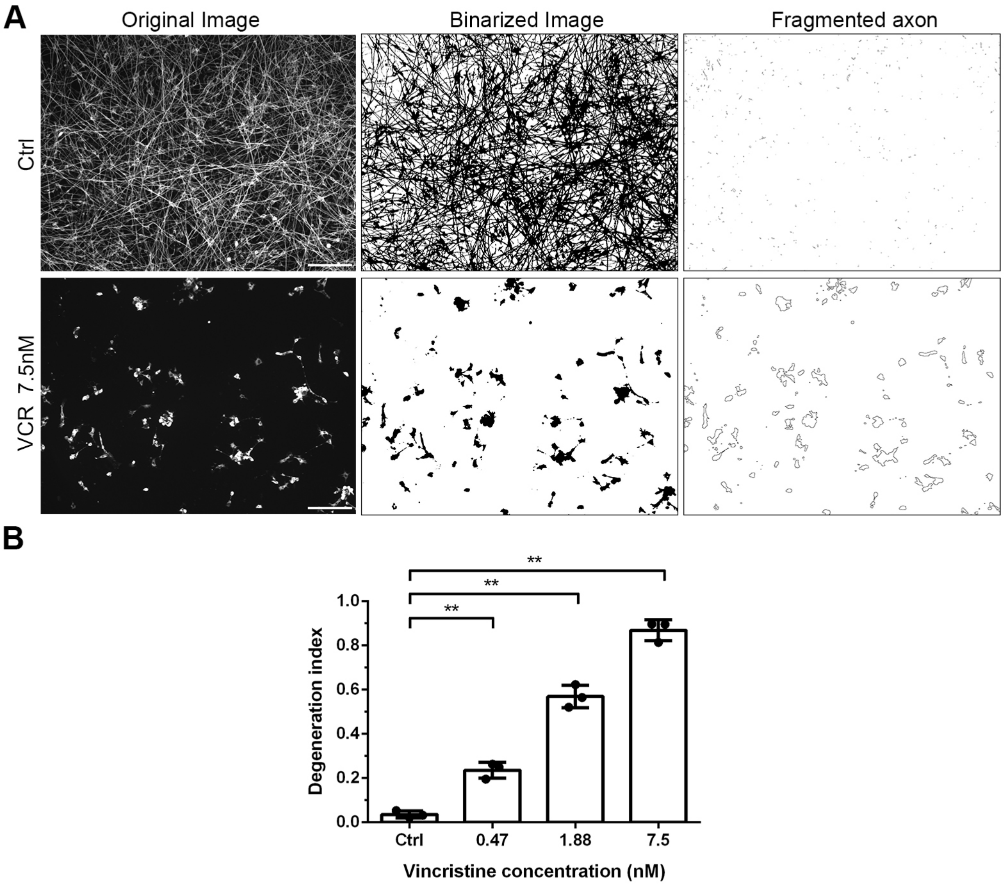

Fig. S3. The axon degeneration index was calculated after vincristine treatment. The area of total axons and fragmented axons was measured using ImageJ, and then the degeneration index was calculated. (A) Representative pictures of original images, binarized images, and fragmented axons from control group and 7.5 nM vincristine for 48 h. The subfigures (original images) of Ctrl and VCR 7.5 nM are reused from Fig. 4A (for quantifying neurite length and neuron number) to demonstrate the quantification of axon degeneration index. Scale bar: 20 µm. (B) The neurons were treated with various concentrations of vincristine (0.47, 1.88, and 7.5 nM) for 48 h. The degeneration index analysis confirmed neurite injury after vincristine treatment. One way ANOVA followed by Tukey's multiple comparisons test, * $p < 0.05$ and ** $p < 0.01$ compared to the control group, n=3.

Disease Models & Mechanisms • Supplementary information

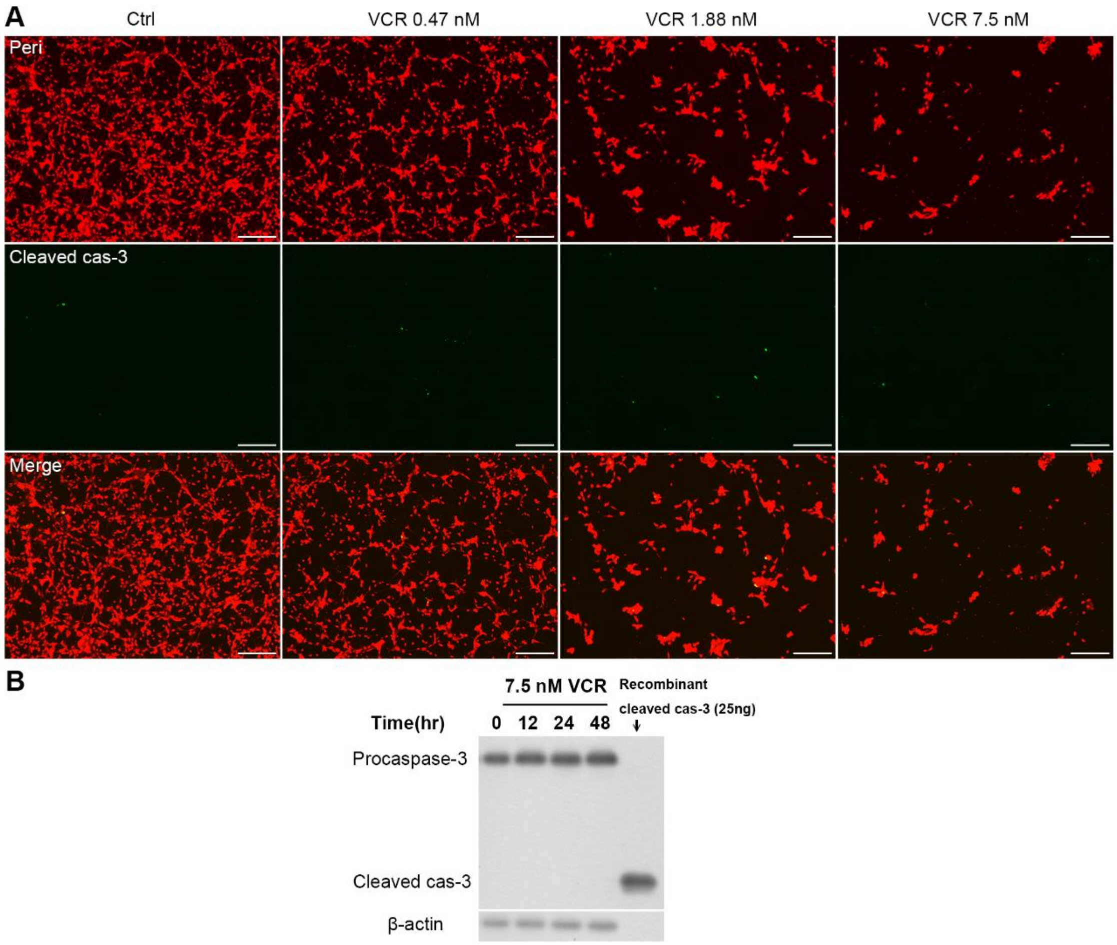

Fig S4. The effects of apoptosis in iPSCs-derived sensory neurons after Vincristine treatment.

iPSC-derived sensory neurons were treated with vincristine and then examined with apoptosis markers (cleaved caspase-3) by immunofluorescence staining and western blotting.

(A) Cells were stained with peripherin (red) and cleaved caspase-3 (green) after being treated with 0.47/1.88/7.5 nM vincristine for 48 h. scale bar = 20 µm.

(B) Cells were treated with 7.5 nM vincristine for 12/24/48 h. Western blot analysis against procaspase-3 and cleaved caspase-3 after vincristine treatment was conducted. Recombinant caspase-3 protein and β-actin were used as positive control and internal control, respectively.

Disease Models & Mechanisms • Supplementary information

Table S1. Primary and Secondary antibodies.

Antibody	Catalogue	Host	Dilution	Manufacturer
**Immunocytochemistry**				
Peripherin	PER	Chicken IgG	1:200	Aves
βIII-tubulin	T5076	Mouse IgG2b	1:1000	Sigma
SMI-32<br>(Neurofilament heavy chain)	SMI-32P	Mouse IgG1	1:10000	Biolegend
Cleaved Caspase-3 (Asp175)	#9661	Rabbit IgG	1:200	Cell signaling
Alexa Fluor® 488<br>anti-mouse IgG<br>Fcγ subclass 1 specific	155-545-205	Goat IgG	1:500	Jackson
Cy™3<br>anti-chicken IgG (H+L)	703-165-155	Donkey IgG	1:500	Jackson
Alexa Fluor® 647<br>anti-mouse IgG<br>Fcγ subclass 2b specific	155-605-206	Goat IgG	1:500	Jackson
Alexa Fluor® 488<br>anti-rabbit IgG	111-545-003	Goat IgG	1:500	Jackson
**Western blot**				
LC3	4108	Rabbit IgG	1:1000	Cell signaling
SQSTM1/p62	ab56416	Mouse IgG	1:1000	Abcam
p-JNK	4668S	Rabbit IgG	1:1000	Cell signaling
JNK	9252	Rabbit IgG	1:1000	Cell signaling
p-p38	9211	Rabbit IgG	1:1000	Cell signaling
P38	ab197348	Rabbit IgG	1:1000	Abcam
p-ERK1/2	9101	Rabbit IgG	1:1000	Cell signaling
ERK1/2	4695	Rabbit IgG	1:1000	Cell signaling
Caspase-3	GTX110543	Rabbit IgG	1:1000	GeneTex
β-actin	A1978	Mouse IgG	1:5000	Cell signaling

Table S2. Sequences of primer.

Gene	Sequence(5' to 3')	Tm (℃)
NANOG	F: AGT CCC AAA GGC AAA CAA CCC ACT TC<br>R: TGC TGG AGG CTG AGG TAT TTC TGT CTC	62
SOX2	F: GGG AAA TGG GAG GGG TGC AAA AGA GG<br>R: TTG CGT GAG TGT GGA TGG GAT TGG TG	56
PRPH	F: ATG GCC GAG GCC CTC AAC CAA GAG<br>R: TAG GCG GGA CAG AGT GGC GTC GTC	56
OCT4	F: GAC AGG GGG AGG GGA GGA GCT AGG<br>R: CTT CCC TCC AAC CAG TTG CCC CAA AC	56
PAX6	F: TCT TTG CTT GGG AAA TCC G<br>R: CTG CCC GTT CAA CAT CCT TAG	62
TUJ1	F: GGC CAA GGG TCA CTA CAC G<br>R: GCA GTC GCA GTT TTC ACA CTC	62
REX1	F: CGC AAT CGC TTG TCC TCA GAG T<br>R: GCT CTC AAC GAA CGC TTT CCC A	58
ASCL1	F: TCC CCC AAC TAC TCC AAC GA<br>R: GCG ATC ACC CTG CTT CCA AA	52
TRPV1	F: GGC TGT CTT CAT CAT CCT GCT GCT<br>R: GTT CTT GCT CTC CTG TGC GAT CTT GT	62
TRKA	F: TCT TCA CTG AGT TCC TGG AG<br>R: TTC TCC ACC GGG TCT CCA GA	62
TRKB	F: AGG GCA ACC CGC CCA CGG AA<br>R: GGA TCG GTC TGG GGA AAA GG	56
TRKC	F: CAC GCC AGG CCA AGG GTG AG<br>R: GAA TTC ATG ACC ACC AGC CA	62
RUNX1	F: CTG CTC CGT GCT GCC TAC<br>R: AGC CAT CAC AGT GAC CAG AGT	64
SHOX2	F: CAG CCA GTT TGA AGC TTG TAG AG<br>R: GAA CCT GAA AGG ACA AGG GCG T	56
RUNX3	F: CAG AAG CTG GAG GAC CAG AC<br>R: GTC GGA GAA TGG GTT CAG TT	50
CGRP	F: TGC CCA GAA GAG AGC CT<br>R: TGA AGG TCC CTG CGG C	52
TAC1	F: TTA CTG GTC CGA CTG GTA CGA C<br>R: CAA AGA ACT GCT GAG GCT TGG G	58

Disease Models & Mechanisms • Supplementary information

RET	F: GAG GAG AGA CTA CTT GGA CCT TG<br>R: GGG GAC AGC GGT GCT AGA AT	64
NFH	F: GCC TGA GGA GAA ACC CAA GAC<br>R: TTT CAG CCT TTT CTG CCT TAG G	64

Disease Models & Mechanisms • Supplementary information
